# Essential role of Rnd1 in innate immunity during viral and bacterial infections

**DOI:** 10.1038/s41419-022-04954-y

**Published:** 2022-06-02

**Authors:** Akhilesh Kumar, Shalabh Mishra, Ashish Kumar, Ashwin Ashok Raut, Seiichi Sato, Akinori Takaoka, Himanshu Kumar

**Affiliations:** 1 Department of Biological Sciences, Indian Institute of Science Education and Research Bhopal, Bhopal, India; 2 Department of Dermatology, School of Medicine, University of California Davis, Sacramento, CA USA; 3 Pathogenomics Lab, ICAR-National Institute of High Security Animal Diseases, Bhopal, Madhya Pradesh India; 4 Division of Signaling in Cancer and Immunology, Institute for Genetic Medicine, Hokkaido University, Sapporo, Japan; 5 WPI Immunology, Frontier Research Centre, Osaka University, Osaka, Japan

**Keywords:** Viral infection, Pattern recognition receptors

## Abstract

Intracellular and cell surface pattern-recognition receptors (PRRs) are an essential part of innate immune recognition and host defense. Here, we have compared the innate immune responses between humans and bats to identify a novel membrane-associated protein, Rnd1, which defends against viral and bacterial infection in an interferon-independent manner. Rnd1 belongs to the Rho GTPase family, but unlike other small GTPase members, it is constitutively active. We show that Rnd1 is induced by pro-inflammatory cytokines during viral and bacterial infections and provides protection against these pathogens through two distinct mechanisms. Rnd1 counteracts intracellular calcium fluctuations by inhibiting RhoA activation, thereby inhibiting virus internalisation. On the other hand, Rnd1 also facilitates pro-inflammatory cytokines IL-6 and TNF-α through Plxnb1, which are highly effective against intracellular bacterial infections. These data provide a novel Rnd1-mediated innate defense against viral and bacterial infections.

## Introduction

Infectious diseases have always been the most significant challenge to human health and a burden on global economies. With the improved sanitation, development of vaccines, and antibiotics, the burden of infectious diseases diminished to low levels or completely disappeared during the 1970s [1], and it was considered that the battle against infections had been won. However, due to the emergence of antibiotic-resistant microbial pathogens and appearance of novel pathogens, the threat of infectious diseases is again rising [1]. The emergence of novel diseases is mainly attributed to zoonosis, a spillover of pathogens from animal hosts to humans [2]. Since the pathogen is utterly new to the host immune system, emerging disease poses an enormous threat to humans, which is best demonstrated by the ongoing coronavirus disease (COVID-19) pandemic caused by SARS-CoV-2. When a novel pathogen infects humans, the only available defense is the hosts’ innate immune system. It is composed of physical defenses, innate immune cells, and a myriad of intracellular and cell surface familes of sensors present in the host cells known as pattern-recognition receptors that recognise broad array of pathogen-associated molecular patterns (PAMPs). Upon recognition, these molecules activate a signalling cascade that culminates in the secretion of innate immune factors such as pro-inflammatory cytokines and type-I and III interferons that act in an autocrine and paracrine manner to induce the production of different proteins that help cells to fight against the invading pathogens. These molecular defense mechanisms are evolutionarily conserved, but transcriptional regulation might differ in different organisms. Bats are considered to be a natural reservoir host for numerous human pathogens, including viruses and bacteria [3–5]. These pathogens have co-evolved with bats; while bat cells support infection and replication of pathogens, they somehow keep the replication in control so that the bat itself rarely exhibits any signs of disease. It is thought that their unique biological and immunological characteristics allow bats to harbour pathogens [6]. Bat cells constitutively express type-I interferons and several other innate immune molecules at higher levels that limit pathogen number and subsequently the development of disease [7]. We compared publicly available human and bat transcriptomic data in the basal state and after virus infection and found that a gene Rnd1 that is not characterised as antiviral is induced more than 200 folds in bat cell lines but not in humans. Additionally, previously it has been reported that Rnd1 is upregulated during Newcastle disease virus infection and interferon treatment in fruit bat (*Pteropus vampyrus*) cells indicating that enhanced Rnd1 expression might be one of the factors behind the tolerant nature of bats against various viral infections [8], therefore, we sought to analyse its role in humans.

Rnd1 belongs to the family of Rho GTPases. Most members of this family function as molecular switches by cycling between a GTP-bound active form and a GDP-bound inactive form. Interestingly, Rnd family proteins in humans lack the GTPase activity and, therefore, always remain in GTP-bound form. Instead of GDP/GTP switch, their activity is regulated by expression, localisation, and phosphorylation. Rnd family proteins include Rnd1, Rnd2, and Rnd3 in humans, and like other Rho GTPases, they also regulate the polymerisation of actin cytoskeleton [9]. Recent studies indicate that these proteins play a crucial role in axon guidance, cell cycle, and tumorigenesis [10]. Here, we report for the first time that Rnd1 is induced in human cells, but to a lesser extent than in bats, the increase in expression is mainly driven by pro-inflammatory cytokine signalling and not interferon signalling. We show that Rnd1 does not just antagonise viral infection but also bacterial infections, although through two different molecular mechanisms.

## Results

### Rnd1 is induced during viral and bacterial infections

**Fig. 1:**
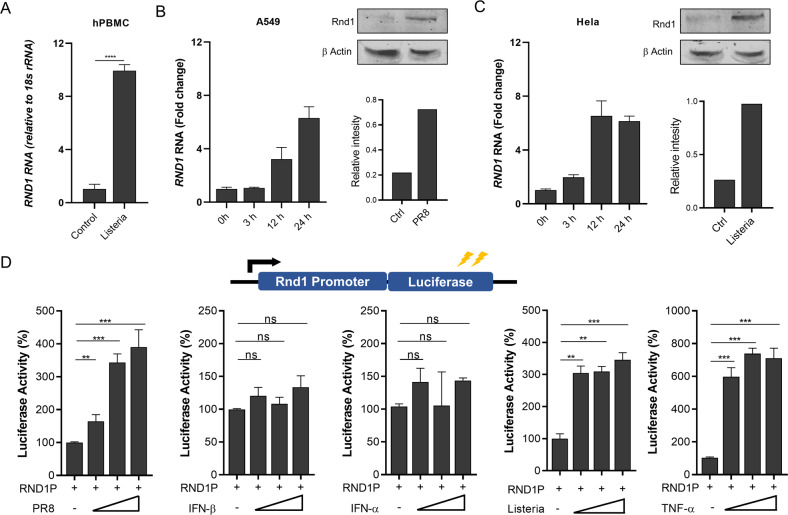
Induction of Rnd1 during viral and bacterial infections. **A** peripheral blood mononuclear cells (PBMCs) were infected with *Listeria monocytogenes* (LM) for 24 h and Rnd1 expression was analysed by RT-PCR analysis, **B** A549 cells were infected with PR8 or **C** Hela cells were infected with LM and expression of Rnd1 was analysed by RT-PCR after 0, 3, 12, and 24 h of infection or by western blotting using specific antibody 12 h post infection. **D** Rnd1 promoter was cloned upstream to luciferase gene. The resulting plasmid (Rnd1P) was transfected into HEK293 cells, and luciferase activity was analysed 12 h after treatment with shown virus, bacteria or purified ligand. Cells were treated with 50, 100, 500 U/ml IFN-β, 100, 500, 1000 U/ml IFN-α, and 25, 50, 100 ng/ml TNF- α or infected with 0.5, 1, or 2 MOI of PR8 or LM. Data are representative of results from three independent experiments. RT-PCR and promoter reporter assay data are means ± SEMs from triplicate samples of a single experiment.

It has been reported that *P. vampyrus* express several atypical ISGs, including Rnd1, that have not been characterised as antiviral genes in humans or murine species [8]. Expression of Rnd1 is significantly increased in bat kidney cells infected with Newcastle Disease Virus (NDV). To further study induction of Rnd1 in bats, we analysed publicly available gene expression data from the NCBI-GEO database. Reanalysis of data indicated that the level of Rnd1 induction varies depending on viruses and bat species; however, this induction was less prominent in humans (Supplementary figure S1A–C). To confirm the induction of Rnd1 in humans, we infected A549 cells with influenza virus H1N1 strain PR8 (PR8) and Hela or peripheral blood mononuclear cells (PBMCs) with *Listeria monocytogenes* (LM) and analysed the expression of Rnd1 (Fig. 1A–C). Similarly, induction of Rnd1 in response to Newcastle disease virus (NDV) was also observed in HEK293 cells (Supplementary fig. S1D). To understand the factors inducing Rnd1 expression, the Rnd1 promoter was cloned upstream to the luciferase gene and analysed luciferase activity after stimulation with PR8, IFN-α, IFN-β, LM and TNF-α. Results suggest that Rnd1 is induced by virus and bacterial infection and pro-inflammatory cytokines but not by Type-I interferons (Fig. 1D).

### Rnd1 suppresses viral and bacterial replication

**Fig. 2:**
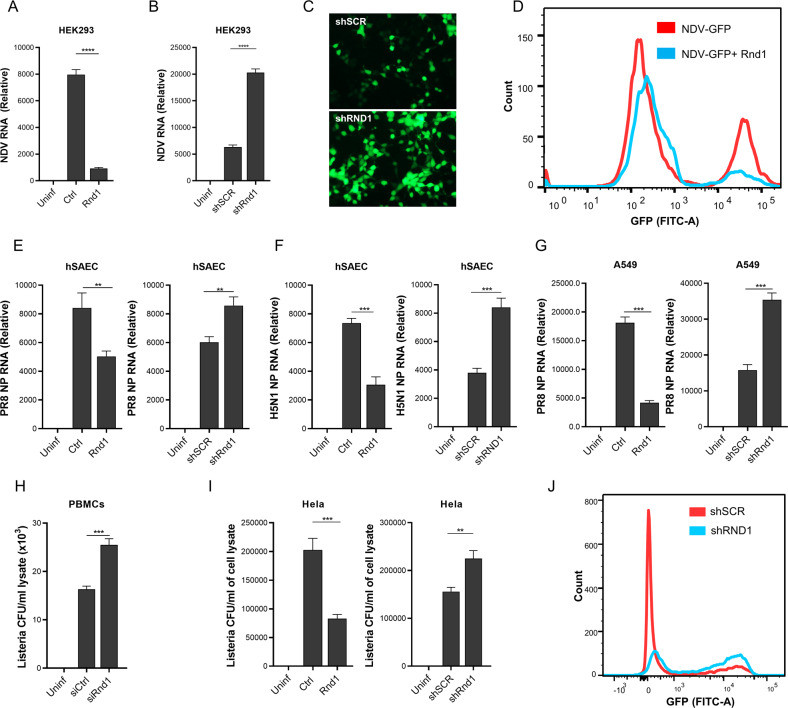
Rnd1 suppress viral and bacterial replications. **A** HEK293 cells were transfected with either empty vector backbone (Ctrl) or Rnd1 expressing plasmids followed by infection with GFP-tagged NDV. 24 h post-infection NDV viral load was analysed in cells by RT-PCR. **B** HEK293 cells were transfected with either control shRNA or shRNA targeting Rnd1, cells were infected with GFP-tagged NDV 24 h post-infection, and NDV viral load was analysed in cells by **B** RT-PCR, **C** epifluorescence microscopy or **D** flow cytometry. Similarly, Rnd1 was either overexpressed or knocked down in primary human (h)SAECs or A549 cells and infected with **E** PR8, **F** H5N1 or **G** PR8. Viral load was analysed by RT-PCR 24 h post-infection. **H** PBMCs were transfected with siRNA targeting RND1, 24 h post-transfection, cells were infected with LM, 24 h post-infection, bacterial load was analysed by colony formation assay. **I** Hela cells were infected with GFP-tagged *L. monocytogens* after Rnd1 was either overexpressed or knocked down. 24 h post-infection, bacterial load was analysed by colony formation assay, or **J** flow cytometry. Data are representative of results from three independent experiments. RT-PCR and colony formation assay data are means ± SEMs from triplicate samples of a single experiment.

As Rnd1 expression is induced during bacterial and viral infection, we tested its role during bacterial and viral infection. We transfected HEK293 cells either with Rnd1 encoding plasmid (pCMV-Flag-Rnd1) or vector backbone (pCMV-Flag-6c) and subsequently infected by NDV (GFP-tagged) and finally analysed for viral load 24 h post-infection, the viral load was significantly reduced (~10 fold) as shown in Fig. 2A. To confirm the role of Rnd1, Rnd1 knockdown cells were prepared by transfecting shRNA targeting Rnd1 (shRnd1) or non-targeting scrambled control (shSCR) (Supplementary fig. S2A). NDV viral load was analysed 24 h post-infection by qRT-PCR, epifluorescence microscopy, or flow cytometry. We found that Rnd1 knockdown significantly enhanced viral load (Fig. 2B–D). Furthermore, we confirmed our observations in primary human small airway epithelial cells (SAEC) using influenza virus vaccine strain PR8 and highly pathogenic influenza virus, a pneumotropic virus. To this end, overexpression and knockdown of Rnd1 were performed as explained above. Cells with overexpression or knockdown of Rnd1 show significantly reduced or enhanced viral load, respectively, as measured by qRT-PCR (Fig. 2E, F). Additionally, similar results were obtained with A549 cells (Fig. 2G). Next, we investigated the effect of Rnd1 overexpression or knockdown on LM infection, an intracellular gram-positive bacteria with GFP-tag. Post-infection the cells were analysed by CFU assay, qRT-PCR using primer specific for HlyQ gene of LM, and cytofluorometric analysis for GFP and found a similar trend as observed with viral infection concluding that Rnd1 significantly suppresses LM multiplication and involve in the protection (Fig. 2H–J and supplementary fig. S2B, C). Collectively, our results suggest that Rnd1 is pivotal for viral and bacterial replication.

### GTP-binding motif of Rnd1 along with Plxnb1 restrict pathogen through induction of pro-inflammatory cytokines

**Fig. 3:**
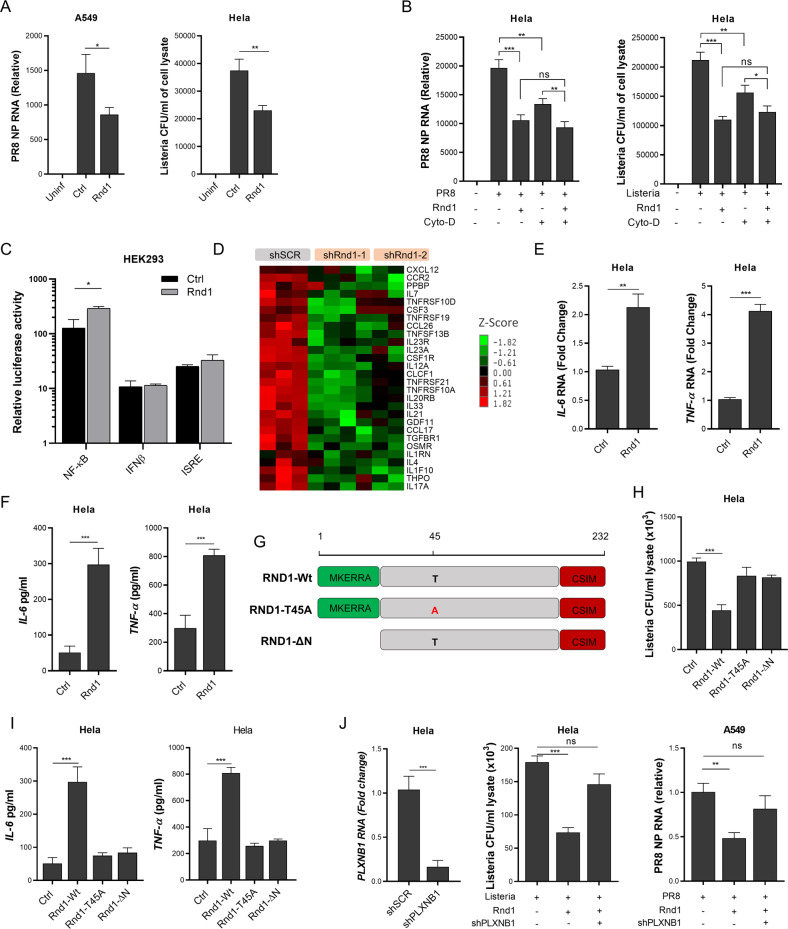
Rnd1 prevents pathogen internalisation and induces pro-inflammatory cytokine. **A** Rnd1 was overexpressed in A549, or Hela cells and were infected with PR8 or *L.monocytogenes*, respectively. In all, 1 h post-infection, cells were analysed for viral load by RT-PCR or bacterial load by colony formation assay. **B** 24 h after transfection of EV or Rnd1 plasmids, cells were treated with cytochalasin D for 30 min and infected either with PR8 (A549) or *L. monocytogenes* (Hela). **C** Rnd1 expression plasmid along with NF-κB, IFNβ, or ISRE luciferase reporter plasmid was transfected into HEK293 cells; after 24 h, relative luciferase activity was measured. **D** Heatmap of dysregulated cytokines upon Rnd1 knockdown in MCF10A cells, data was obtained after reanalysing publicly available data from NCBI-GEO (GSE43885). IL-6 and TNF-α expression was analysed after Rnd1 overexpression in Hela cells by **E** RT-PCR and **F** ELISA. **G** Schematic representation of the wild type and mutant Rnd1. **H** Analysis of *L. monocytogenes* bacterial load and **I** IL-6 and TNF-α expression upon overexpression of wild type and mutant Rnd1 in Hela cells. **J** Analysis of *L. monocytogenes* and PR8 load upon Rnd1 overexpression in Plxnb1 knocked down cells. Data are means ± SEMs from triplicate samples of a single experiment and are representative of results from three independent experiments.

Previously it has been reported that Rnd1 is localised on the plasma membrane, and therefore, it was speculated that Rnd1 might be crucial in internalisation during viral and bacterial infection. To study this hypothesis, A549 and Hela cells expressing Rnd1 were infected with PR8 or LM, respectively, and viral and bacterial load were analysed 1 h post-infection. We observed that Rnd1 overexpression decreased viral and bacterial load even after 1 h post-infection, suggesting Rnd1 may affect microbial internalisation (Fig. 3A). Notably, it has been suggested that influenza viruses take approximately 1 h to deliver viral RNA into the nucleus and take 3 h to start the first replication cycle [11, 12]. Similarly, LM also takes more than 1 h to start replication after getting inside the cell, and its doubling time is approximately 40 min [13]. Therefore, any difference in viral or bacterial load observed during this experiment should reflect a defect in the internalisation process. Both viruses and bacteria use cellular cytoskeleton to internalise, and Rnd1 also regulates actin dynamics. To understand the link between Rnd1 and actin dynamic during viral and bacterial infection, we used a potent inhibitor of actin polymerisation, cytochalasin D. Cells which were initially expressing empty vector or Rnd1 were pre-treated with 20 µM cytochalasin D for 30 min, followed by infection with PR8 or LM. Results show that both cytochalasin D and Rnd1 individually decrease internalisation. These results also held true when Rnd1 was knocked down using shRNA (Supplementary fig. S3A, B). However, Rnd1 and cytochalasin D did not produce synergistic effects when both treatments were given simultaneously (Fig. 3B), indicating that Rnd1 had some different mechanism to restrict viral and bacterial entry into the cell than the reorganisation of actin cytoskeleton. Notably, the cytochalasin D or RND1 shRNA transfection along with cytochalasin D treatment did not affect cell health as analysed by MTT assay (data not shown).

To explore the possible mechanism for Rnd1-mediated viral and bacterial restriction, we tested the ability of Rnd1 to induce Type-I interferon or pro-inflammatory cytokine. The Rnd1 was overexpressed along with NF-κB, IFNβ, and ISRE promoter, and promoter activity was analysed by dual luciferase assay. Rnd1 overexpression did not promote IFNβ and ISRE but induced NF-κB promoter activity (Fig. 3C). To confirm these observations, we reanalysed publicly available transcriptomic data involving Rnd1 knockdown from the NCBI-GEO database (GSE43885) and found that Rnd1 knockdown substantially decreased the expression of several NF-κB regulated inflammatory cytokine genes, as shown in Fig. 3D, and also downregulate cytokines and inflammatory response pathways (Supplementary Fig S4), which was in agreement to the results of luciferase assay. Next, we overexpressed Rnd1 in Hela cells followed by infection with LM and tested relative expression by RT-PCR or measured indicated cytokine by ELISA. The results suggest that Rnd1 promotes IL-6 and TNF-α production (Fig. 3E, F). Moreover, Rnd1 did not reduce the bacterial load if IL-6 was blocked, and Rnd1 overexpressing cells produced more reactive oxygen species during listeria infection, which is a possible effect of elevated TNF- α (Supplementary fig. S4B, C), further confirming that Rnd1 reduced bacterial load by inducing IL-6 and TNF- α.

To gain molecular insight into the suppression of viral and bacterial load in Rnd1 expressing cells, we next investigated the role of various domains of Rnd1. Previously, it has been shown that the N-terminal domain is responsible for translocation of Rnd1 to the plasma membrane and C-terminal domain is essential for farnesylation and attachment with lipids in membrane; the intermediate region is essential for GTP binding, and essential for the Rnd1 activity, and T45A mutant abolishes GTP binding and acts as a dominant-negative. Therefore, various mutants were created, as shown in Fig. 3G. The cells overexpressing Rnd1-T45A or Rnd1-ΔN were unable to restrict bacterial infection (Fig. 3H and supplementary fig. S5A) and promote IL-6 and TNF-α production (Fig. 3I), indicating that both spatial localisation to plasma membrane and GTP binding is necessary for its function. Previously, it has been reported that Rnd1 binds to Rho GTPase binding domain (RBD) on several plexin proteins with preference to Plxnb1 [14, 15]. Plexins are semaphoring receptors consisting of an RBD, a GTPase-activating protein (GAP), and a GTPase binding domain. Their role as receptors for immune guidance molecules has recently been identified. Reports indicate that Type-B plexins are abundant in lung epithelium [16], and their activation induces pro-inflammatory cytokine production [17, 18]. Although the exact mechanism is unclear, it is thought that Rnd1 interaction with plexin-B1 regulates Rho GTPases either through their intrinsic RhoGAP activity or through PDZ-RhoGEF to induce cytokine expression [15, 19, 20]. Upon activation, Plxnb1 was observed to transiently decrease cellular RhoA-GTP levels in adherent cells [21]. Additionally, the inhibition of RhoA/ROCK signalling was found to extend the duration of p65-DNA binding, IκBα phosphorylation and IKKβ activity following LPS treatment. Therefore, we analysed the effect of Plxnb1 knockdown on RhoA activation and NF-kB promoter activity in Rnd1 overexpressing cells. As expected, we found that Rnd1 overexpression decreased RhoA-GTP and induced NF-κB activity, which was abolished when Plxnb1 was knocked down (Supplementary fig. S5B, C). Finally, to analyse if inhibition of bacterial or viral infection by Rnd1 is dependent on Plxnb1, we performed shRNA-mediated knockdown of Plxnb1 in Hela or A549 cells (Plxnb1 knockdown did not affect Rnd1 overexpression, data not shown) and found that Rnd1 overexpression could not reduce bacterial or viral load (Fig. 3J). Collectively, these results suggest that N-terminal and GTP-binding motif of Rnd1 along with Plxnb1 are essential for the induction of inflammatory cytokines through transcription factor NF-κB and confers viral and bacterial restriction.

### Rnd1 dysregulates calcium signalling pathway to prevent virus entry

**Fig. 4:**
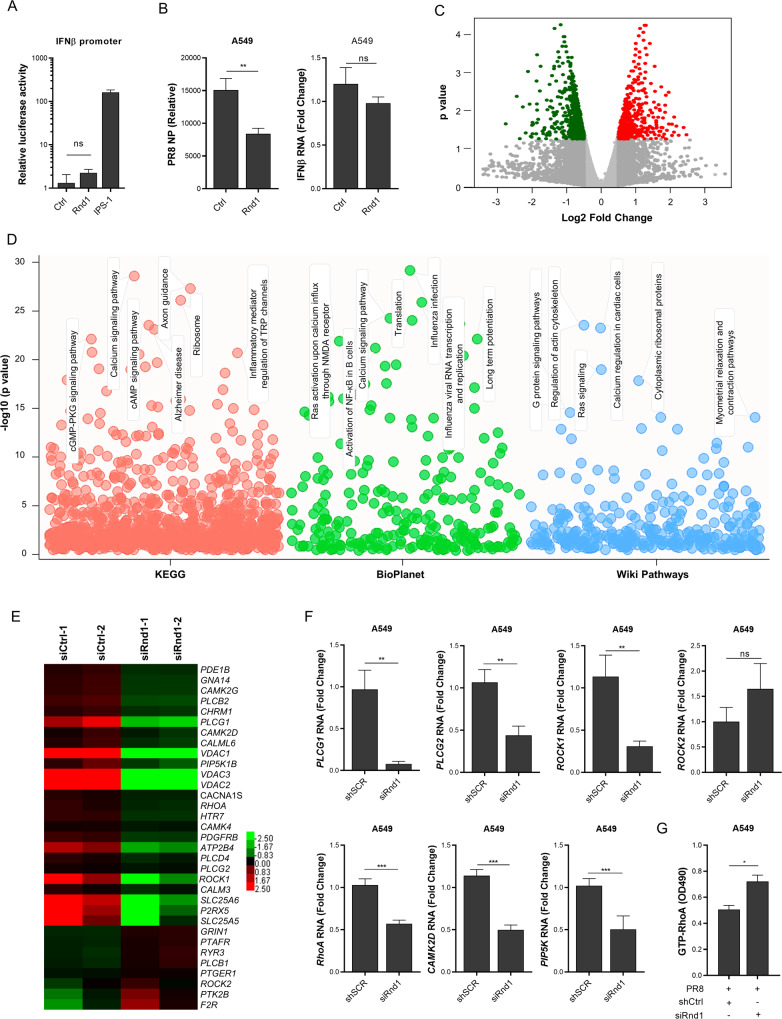
Rnd1 knockdown disrupts the calcium signalling pathway. **A** HEK293 cells were transfected with IFNβ reporter plasmid along with either the vector backbone (Ctrl), Rnd1 or IPS-1 expressing plasmids followed by PR8 infection; IPS-1 was used as a positive control. **B** Rnd1 was overexpressed in A549 cells and followed by disruption of Type-I interferon signalling using IFNAR2 blocking antibody. PR8 viral load and IFNβ were analysed 24 h post infection. **C** Volcano plot indicating down-regulated (green) and upregulated (red) genes. **D** Pathway analysis indicating downregulated pathways from three different databases- KEGG, BioPlanet and Wiki Pathways, top significant pathways have been highlighted. **E** Heatmap of calcium signalling pathway genes that are dysregulated by Rnd1 knockdown. **F** Analysis of selected genes after knockdown from heatmap by RT-PCR to verify the results of RNA-Seq experiment. **G** Analysis of RhoA activation after siRNA-mediated knockdown of Rnd1 in A549 cells. RT-PCR and promoter-reporter assay data are means ± SEMs from triplicate samples of a single experiment and are representative of results from three independent experiments. RNA-Seq data are representative of a single experiment with two replicates.

To understand the molecular mechanism for PR8 restriction, we first tested the effect of Rnd1 on IFNβ promoter activity by luciferase assay during PR8 infection and found that Rnd1 doesn’t induce IFNβ promoter activity (Fig. 4A). To verify PR8 viral load reduction upon Rnd1 overexpression is an interferon-independent phenomenon; first, Rnd1 was overexpressed in A549 cells that were pre-treated with IFNAR2 blocking antibody to disrupt Type-I interferon signalling (Fig. 4B), and subsequently infected with PR8. Additionally, we used interferon deficient Vero E6 cells overexpressing or knockdown of Rnd1. Cells overexpressing Rnd1 show significantly low viral load, whereas knockdown cells show substantially higher viral load (Supplementary fig. S5E, F), suggesting type I interferon-independent viral restriction. Next, to gain further insights into the effect of Rnd1 on the transcriptional landscape of cells during virus infection, we performed global transcriptomic analysis by RNA-Seq. We knockdown Rnd1 using gene-specific siRNA and infected cells with H5N1 for 12 h. To test the knockdown efficiency of Rnd1 and H5N1 viral load inside cells RT-PCR was performed, as shown in supplementary fig. S5G. Total RNA was isolated, and mRNA was subjected to RNA-Seq analysis (Fig. 4C). Pathway analysis of dysregulated genes indicates downregulation of Axon guidance, Ras signalling, translation, Alzheimer’s disease, and influenza infection pathways.

Interestingly, we noticed that the calcium signalling pathway was also downregulated (Fig. 4D and supplementary fig. S6A). Several essential genes involved in calcium signalling like VDAC, Rho-kinase, CAM kinases and phospholipase C were downregulated (Fig. 4E). To validate RNA-Seq data, expression of selected genes involved in calcium signalling pathway such as PLCG1, ROCK1, RHOA, etc., was also analysed by qRT-PCR analysis (Fig. 4F) suggesting that calcium-associated pathways may be critical for PR8 restriction. However, most of these dysregulated molecules are either kinases, GTPases, or ion channels and therefore exist in two forms active and inactive. Analysing their RNA levels alone could not confirm their effect on Ca^2+^ oscillations. Consequently, we analysed the activation of RhoA, which is a major upstream regulator of Ca^2+^ oscillations [22]. Although Rnd1 knockdown decreased expression of RhoA, it increased the active GTP-bound form of RhoA, which was in agreement with previous findings that Rnd1 inhibits RhoA (Fig. 4G).

### Rnd1 prevents virus entry into cells by disrupting cytoplasmic calcium oscillations

**Fig. 5:**
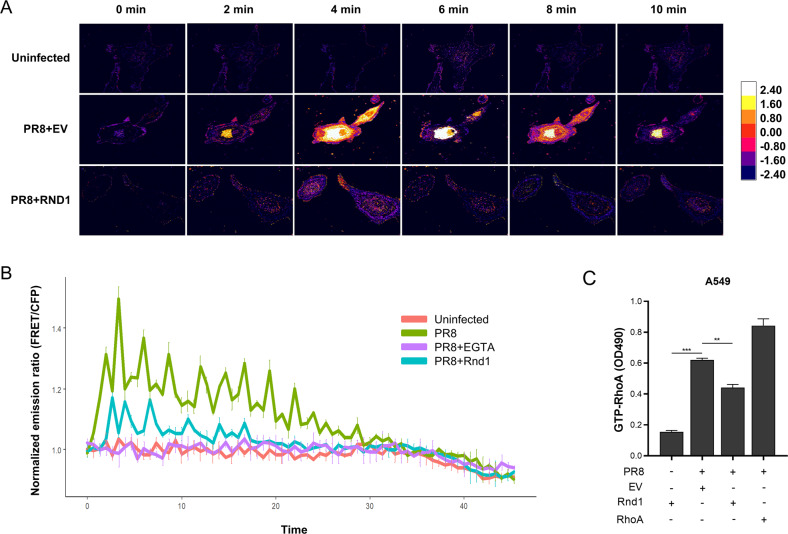
Rnd1 disrupts cytoplasmic calcium oscillations. **A** Plasmid expressing Rnd1 or EV was transfected into A549 cells along with YC3.6 plasmids. Microscopic images were recorded for CFP (480 nm) and YFP (530 nm) at intervals of 60 s. Images were analysed by FRETanalyzer plugin in ImageJ software. **B** As an indicator of intracellular calcium levels, FRET/CFP lvel was plotted against time. **C** A549 cells were transfected either with plasmid expressing Rnd1, RhoA, or EV followed by infection with PR8. Rho A activation was analysed using colorimetric G-LISA RhoA activation assay. Data are means ± SEMs from triplicate samples of a single experiment and are representative of results from three independent experiments.

Recently, it has been reported that Calcium ion (Ca^2+^) is a crucial factor in influenza virus internalisation and successful infection, and it activates a signalling axis comprising RhoA, phospholipases, phosphatidylinositol 4-phosphate 5-kinase and Rho-kinases. It has been demonstrated that during infection, influenza virus induces cytosolic Ca^2+^ oscillations in host cells, and prevention of these ion oscillations markedly reduces virus entry into the cell [22]. Additionally, it has been shown that a similar signalling mechanism based on intracellular Ca^2+^oscillations has been implicated in several other viruses and bacteria, including LM infection [23–25]. Rnd1 has been reported to antagonise RhoA function by directly inhibiting its activation by GTP binding [26–28]. Therefore, we analysed fluctuations in intracellular calcium levels using fluorescence resonance energy transfer (FRET) based on the calcium sensor yellow chameleon (YC 3.6). When not bound to Ca^2+^ YC has emission maxima at 480 nm; upon binding calcium, the emission maxima shift to 530 nm due to FRET, and the ratio 530/480 nm emission intensities give an estimate of available intracellular Ca^2+^. Rnd1 overexpression damped the calcium oscillation waves (Fig. 5A, B). Next, we tested the activation of RhoA and found that Rnd1 overexpression decreased the GTP-bound active form of RhoA during PR8 infection (Fig. 5C). We have shown that RhoA inhibition by Rnd1 is Plxnb1 dependent (Supplementary fig. S5B). Therefore, we analysed if Plxnb1-Rnd1 interaction is also required for the inactivation of intracellular calcium oscillation by Rnd1. Rnd1 failed to reduce calcium levels when Plxnb1 was knocked down (Supplementary fig. S5D). Overall, these results confirm that Rnd1 reduces influenza internalisation by Plxnb1-dependent RhoA inactivation, which is necessary for the activation of calcium signalling during virus entry into the host cell.

### In-vivo Rnd1 knockdown enhances viral and bacterial infection

**Fig. 6:**
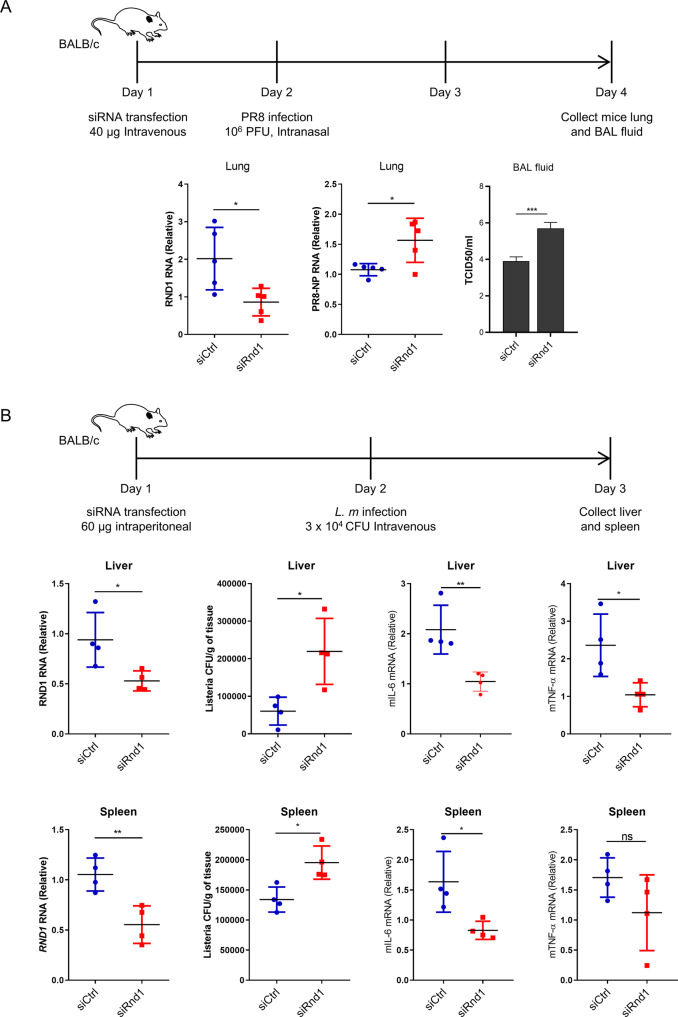
Rnd1 knockdown promotes viral and bacterial infection in-vivo. **A** Mice were intravenously injected with siRNA complexed with in-vivo jetPEI for knockdown of mRnd1. In all, 24 h later, mice were infected with PR8, and after 48 h, lung tissue and BAL fluids were collected. Knockdown efficiency in lung tissue was analysed by RT-PCR followed by analysis of viral load by RT-PCR in lung tissue and TCID50 assay in BAL fluid. **B** For delivery of siRNA to liver and spleen mice were intra-peritoneally injected with siRNA complexed with in-vivo jetPEI. In all, 24 h after transfection, mice were infected intravenously with *L. monocytogenes*. 24 h post-infection, mice were euthanized, and spleen and liver were collected, and knockdown efficiency was confirmed using RT-PCR. Tissues were homogenised, and *L. monocytogenes* load was analysed by colony formation assay. RT-PCR was used to analyse mIL-6 and mTNF-α RNA relative to mGAPDH RNA. Data are means ± SEMs from triplicate samples of a single experiment and are representative of results from three independent experiments.

To confirm our findings in-vivo, we performed siRNA-mediated knockdown of Rnd1 in BALB/c mice. The siRNA was complexed with in vivo-jetPEI and was injected intravenously for lung transfection and intraperitoneally for liver and spleen transfection. Mice were infected intranasally with 1 × 10^6^ PFU of influenza PR8 virus, and lung tissue and bronchoalveolar lavage fluid were collected 2 days after infection (Fig. 6A). For LM infection, mice were infected with 3×10^4^ bacteria intravenously, and spleen and liver tissue were collected after 1 day. We found that Rnd1 knockdown led to increased PR8 viral load in the lungs and BAL fluid (using TCID50 assay) and LM infection in the liver and spleen (Fig. 6B), suggesting that our in vivo results are consistent with in vitro findings.

## Discussion

The classic members of Rho GTPase family have been the subject of numerous studies, whereas atypical molecules like Rnd family proteins have been less explored. While Rnd3 has ubiquitous tissue expression, Rnd2 is present in testis, and Rnd1 is expressed in the liver and several brain tissues and at lower levels in the lungs (Supplementary fig. S7A) [29]. Members of this family are known to play a pivotal role in the regulation of actin cytoskeleton, cell adhesion, cell cycle, and exon guidance through counter activation of Rho family proteins through activation of intrinsic GTPase of p190 RhoGAP [9]. In bats, Rnd proteins are induced upon virus infection or type I interferons stimulation, although its function in human host in immunity is not known. Rnd proteins modulate binding to plexin B1 and modulate downstream semaphorin-mediated signalling. Recent studies have demonstrated their role in innate immunity, including the recruitment and migration of immune cells, modulation of the function and phenotype of myeloid cells, regulation of proinflammatory cytokines and immune cell survival [30]. Additionally, SEMA4A, a ligand to plexin B1, indirectly co-stimulates T cell activity [31]. Therefore, we thought that Rnd1 might have a housekeeping role in host defense. We found that Rnd1 is upregulated during viral and bacterial infections, and the induction is driven by pro-inflammatory cytokines rather than type I interferons like in bats. In human, Rnd1 is present in cells in constitutively active form; therefore, even small changes in their expression might have profound downstream effects. We found that Rnd1 acts as a negative regulator for RhoA by activating Plxnb1 signalling. Inhibition of RhoA by Rnd1 potentiates NF-kB signalling leading to induction of pro-inflammatory cytokines which is responsible for the decrease in bacterial load after Rnd1 overexpression. However, we also observed that inhibition of RhoA also directly prevents pathogens’ internalisation by inhibiting the oscillations in intracellular calcium ion levels. Interestingly, other members of the Rnd family, Rnd2 and Rnd3 did not affect viral replication upon shRNA-mediated knockdown (Supplementary fig. S7B), which could be explained by very low expression of Rnd2 in lung tissues [9] and the inability of Rnd3 to bind to PlexinB and inhibit RhoA [32]. Finally, we verified the relevance of these results in-vivo by knocking down Rnd1 in mice followed by infection with PR8 or Listeria. We could not generate Rnd1 knockout A549 cells, which would have further confirmed the importance of Rnd1 in innate immune responses.

Overall, we have shown for the first time that Rnd1, which was previously reported to be an atypical interferon-inducible gene (ISG) in *P. vampyrus*, is an essential innate immune factor in humans. It defends cells from invading viral and bacterial pathogens by two mechanisms, inducing pro-inflammatory cytokines and inhibiting intracellular calcium waves. Rnd1 is highly conserved between humans and bats (Supplementary fig. S7C), and therefore findings of this study could also shed light on why Rnd1 is upregulated *P. vampyrus* cells in response to virus infection. Rnd1 could be one of the factors that make bats such an excellent reservoir host for various pathogens which are fatal to humans. However, it is also important to note that the expression of Rnd1 as an ISG has not been verified in other bat species. Studies of bat immune response to pathogens have been very limited and are complicated by the high degree of species-specific adaptations in interferon responses [33]. Our study highlights the importance of studying innate immune responses in bats to understand their ability to tolerate microbial pathogens that are otherwise lethal in other mammals. Insights from such studies could guide new strategies to identify novel drug targets in humans and other spillover species.

## Material and methods

### Reanalysis of publicly available data from NCBI-GEO database

Raw sequencing data (fastq files) were transferred from NCBI-GEO database to Galaxy at usegalaxy.org. for further processing and analysis [34]. Data were quality checked, cleaned if necessary and mapped to the reference genome using HISAT2. Cufflinks was used to assemble the aligned RNA-Seq reads into transcripts and estimate the normalised abundance of the assembled transcripts as fragments per kilobase per million (FPKM) [35]. Cuffmerge was used to merge together several Cufflinks assemblies. A merged gtf file produced by cuffmerge was provided as an input to Cuffdiff along with alignment files created by TOPHAT2 for differential analysis between two samples. Various R packages were used for visualisation of expression and differential expression results. Cluster 3.0 and TreeView 1.1.6 [36] were used for making heat maps.

### Quantitative real-time reverse transcription-PCR

Total RNA was extracted using TRIzol reagent (Invitrogen) and was used to synthesise cDNA with the iScript cDNA synthesis kit (Bio-Rad) according to the manufacturer’s protocol. Gene expression was measured by quantitative real-time PCR using gene-specific primers (Supplementary material, table 1) and SYBR green chemistry (Bio-Rad).

### Cells and transfection

A549 human alveolar basal epithelial cells (Cell Repository, NCCS, India), HEK293 human embryonic kidney cells (ATCC CRL-3216) and HeLa cervical cancer cells (Cell Repository, NCCS, India) were cultured in Dulbecco’s modified Eagle’s medium (DMEM) supplemented with 10% fetal bovine serum (FBS) and 1% penicillin-streptomycin. Human small airway epithelial cells (hSAECs) were cultured and maintained as per the manufacturer’s instruction (Lonza). Transfection of DNA, siRNA, or poly I:C in HEK293 cells was performed using Lipofectamine 2000 (Invitrogen), while Lipofectamine 3000 (Invitrogen) was used for A549 and hSAECs.

### Viruses and infection

Influenza virus (strain PR8, A/PR8/H1N1), NDV (strain LaSota) or H5N1 (A/duck/India/02CA10/2011) viruses were used in this study. For infection cells were washed with SF-DMEM and infected with virus dispersed in SF-DMEM. After 1 h, cells were washed and incubated with DMEM supplemented with 1% FBS. Replacement media for PR8 infection was additionally supplemented with l-1-Tosylamide-2-phenylethyl chloromethyl ketone (TPCK)-treated trypsin (1 μg/ml). All experiments related to HPAIV (H5N1) were performed using single viral stocks in biosafety level 3 (BSL3) laboratories at the National Institute of High Security Animal Diseases (NIHSAD). NDV-GFP was kindly gifted by Dr. Peter Palese, Icahn School of Medicine at Mount Sinai, New York, USA.

### *L. monocytogenes* infection and Colony formation assay

GFP-tagged L. monocytogenes (LM) strain EGD was a kind gift from Dr. Pascale Cossart, INSERM, Paris, France. Bacteria were grown in brain heart infusion media to an optical density at 600 nm of 0.8. Bacteria were diluted in SF-DMEM. In all, 3 × 10^5^ Hela cells were seeded per well of six-well plates in antibiotic-free complete DMEM. The next day, cell monolayer was washed with SF-DMEM and incubated with bacterial suspension at approximately 50 multiplicity of infection for 1 h. Cells were washed with PBS, and extracellular bacteria was neutralised by adding complete DMEM supplemented with 25 µg/ml gentamicin (Sigma-Aldrich). After appropriate incubation cells were lysed by adding BHI media supplemented with 0.1% tritonX100. The resulting suspension was serially diluted, and 100 µL was spread on a BHI agar plate. 24 h later the number of bacterial colonies was counted to calculate the number of LM CFU/ml.

### Measurement of viral titre

MDCK cells were infected with serial dilutions of BAL fluid containing the virus and washed with PBS after 1 h of infection. For the 50% tissue culture infective dose (TCID50) assay, cell monolayers were fixed and stained with 1% crystal violet after 96 h of infection, and the TCID50 was calculated based on the Reed and Muench method [37].

### Luciferase reporter assay

Rnd1 promoter was cloned into pGL3-Basic vector upstream to luciferase gene using HindIII and KpnI enzyme sites to make Rnd1 luciferase reporter plasmid. HEK293T cells were seeded at 60–70% confluency into a 24-well plate and transfected with 25 ng pRL-TK plasmid (control) and 200 ng luciferase reporter plasmid (NC). The cells were lysed at 24 h post-transfection, and luciferase activity was measured in total cell lysate using a Glomax multi+ detection system (Promega) using Dual-Glo luciferase assay kit (Promega, E2920).

### Enzyme-linked immunosorbent assay

The culture supernatants were harvested and were analysed by specific Enzyme-linked immunosorbent assay (ELISA) kits according to the manufacturer’s instructions to determine the amounts of TNF-α (555212, Becton Dickinson) and IL6 (555220, Becton Dickinson) secreted by the cells. RhoA activation was measured by RhoA G-LISA Activation Assay Kit (BK124, Cytoskeleton, Inc.) and IκBα phosphorylation was estimated using PathScan® Phospho-IκBα (Ser32) Sandwich ELISA Kit (7355, Cell Signaling Technology).

### Immunoblotting analysis

Cell monolayer was washed with PBS, and cells were harvested after 36 h of infection with standard ice-cold cell lysis buffer supplemented with 1X protease inhibitor cocktail (obtained from Sigma, Aldrich). Cells were collected and subjected to western blotting analysis as previously described [38, 39]. Immunoblotted nitrocellulose membrane was imaged with LI-COR system. Anti–Rnd1 (HPA077800, raised in rabbit) and anti β-actin (A1978, raised in mouse) (6956, raised in mouse) antibodies were obtained from Sigma-Aldrich. Anti-p65 (6956, raised in mouse) was procured from Cell Signaling Technology. IR dye-labelled anti-Rabbit and anti-Mouse IgG (secondary antibody) were purchased from LI-COR. Densitometry analysis was performed by Image J (Fiji) software.

### Epifluorescence microscopy

Cells were seeded in six-well plates at an approximate density of 60–70%. 24 h later, cells were transfected with 1500 ng of shRNA targeting Rnd1. In all, 48 h after transfection, cells were washed with serum free (SF) DMEM and incubated with 1 MOI of NDV-GFP virus in SF-DMEM for 1 h. Cell monolayer was washed with PBS, and media was replaced with complete DMEM. The next day cells were imagined using a ×20 objective on a Zeiss AXIO Vert. A1 inverted microscope.

### Analysis of FRET

In all, 1 × 10^5^ cells were grown on a glass coverslip in 35 mm dishes. For performing live imaging cells edges of a glass slide were covered with parafilm and coverslip containing cells was inverted and put on a glass slide. The space between coverslip and glass slide was filled with cell culture media to keep cells alive. Images of cells were taken with the help of ×63 objective on an ApoTome microscope (ZEISS). For each field of view, donor alone, acceptor alone, and FRET channel micrographs were captured. These confocal micrographs were analysed by FRET and Colocalization analyzer plugin in ImageJ. FRET/CFP normalised emission ratio was plotted against time using ggplot2 R package. For endpoint analysis of intracellular calcium levels, cells were cultured and infected in 96 well black assay plates. Fluorescence intensities were analysed using Eon Microplate Spectrophotometer (Agilent Technologies).

### Flow cytometry

Cells were seeded in six-well plates at approximate density of 60–70%. In all, 24 h later, cells were transfected with 1500 ng of shRNA targeting Rnd1. In all, 48 h after transfection, cells were washed with serum free (SF) DMEM and incubated with 1 MOI of NDV-GFP or 50 MOI of LM in SF-DMEM for 1 h. Cell monolayer was washed with PBS, and media was replaced with complete DMEM. The next day cells were detached using trypsin-EDTA solution and fixed with 4% paraformaldehyde for 5 min.

### RNA-Seq data analysis

Total RNA isolated using TRIzol reagent was processed to prepare cDNA libraries using TruSeq technology according to the manufacturer’s instructions (Illumina, San Diego, CA). Libraries were sequenced using Illumina NextSeq 500, with a read length of 51 bp, by Bencos Research Solutions Pvt. Ltd., Mumbai, India. Assessment of read quality of row data was done using FastQC. Trimmomatic was used to remove Illumina adaptors, and quality filtering of reads was done by the sliding-window approach. Data was quality checked, cleaned if necessary, and mapped to the reference genome using HISAT2. Cufflinks was used to assemble the aligned RNA-Seq reads into transcripts and estimate the normalised abundance of the assembled transcripts as fragments per kilobase per million (FPKM) [35]. Cuffmerge was used to merge together several Cufflinks assemblies. A merged gtf file produced by cuffmerge was provided as an input to Cuffdiff along with alignment files produced by TOPHAT2 for differential analysis between two samples. Various R packages were used for visualisation of expression and differential expression results. Cluster 3.0 and TreeView 1.1.6 [36] were used for making heat maps.

### In-vivo experiments

In total, 6–8-week-old BALB/c mice were intravenously injected with siRNA complexed with in-vivo jetPEI for knockdown of mRnd1. Mice were randomly allocated for injection of either siCtrl or siRnd1. In all, 24 h later, mice were infected with PR8, and after 48 h, lung tissue and BAL fluids were collected. Knockdown efficiency in lung tissue was analysed by RT-PCR followed by analysis of viral load by RT-PCR in lung tissue and TCID50 assay in BAL fluid. For delivery of siRNA to liver and spleen mice were intra-peritoneally injected with siRNA complexed with in-vivo jetPEI. In all, 24 h after transfection, mice were infected intravenously with *L. monocytogenes*. In all, 24 h post-infection, mice were euthanized, and spleen and liver were collected, and knockdown efficiency was confirmed using RT-PCR. Tissues were homogenised, and L. monocytogenes load was analysed by colony formation assay. RT-PCR was used to analyse mIL-6 and mTNF-α RNA relative to mGAPDH RNA.

### Statistical analysis

All experiments were performed with appropriate control samples or mock-transfected samples. Experiments were performed twice or thrice independently of each other. All the data were analysed using GraphPad Prism software for statistical significance. The student’s two-tailed unpaired t test was performed for analysis of statistical significance between two groups. For in-vivo experiments, sample size was chosen based on effect size obtained from a pilot experiment of 3 mice per group. No animals were excluded from the study.

## Supplementary information

Supplementary figure and table

Original Data File

Original Data File

Original Data File

reproducibility checklist

Original Data File

Original Data File

Original Data File

Original Data File

Original Data File

Original Data File

Original Data File

Original Data File

Original Data File

Original Data File

Original Data File

Original Data File

## Data Availability

RNA-Seq data have been submitted to the Gene Expression Omnibus under accession number GSE185216.
