# Supplementary material for: Essential role of Rnd1 in innate immunity during viral and bacterial infections
Source: Cell Death Dis. 2022 Jun 2;13(6):520. doi: 10.1038/s41419-022-04954-y (PMC9161769; doi:10.1038/s41419-022-04954-y)
Supplement: Supplementary file 1 — Supplementary figure and table [file 41419_2022_4954_MOESM1_ESM.pdf]

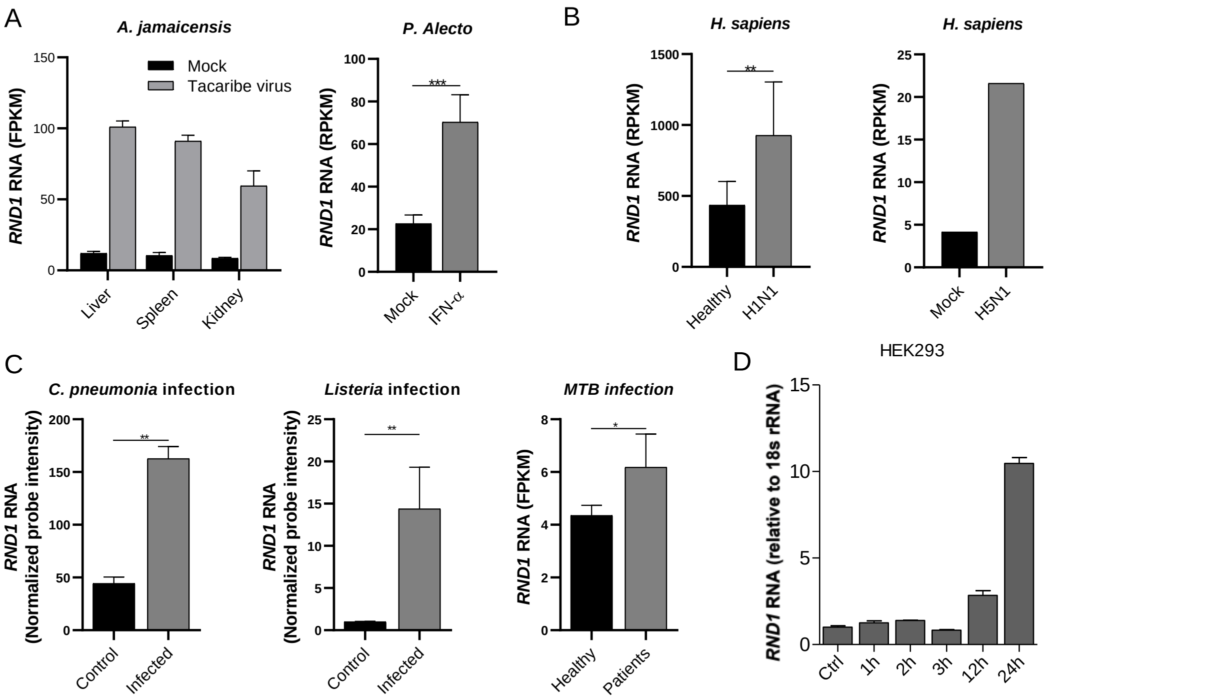

Supplementary Figure S1. Expression of Rnd1 in fruit bats (A) A. jamaicensis (Jamaican Fruit bat) infected with Tacaribe virus (GSE75771) and P. alecto (Black flying fox) cells induced with Type-I interferon for 24 hours (GSE102296) and (B) H1N1 infected lung tissue samples (GSE163959) or human ATII cells infected with H5N1; A/Chicken/Vietnam/0008/04 virus for 24 hours (GSE119767). (C) Induction of Rnd1 expression during bacterial infections, *M. tuberculosis* infected patients (GSE148171), human dendritic cells infected with *Chlamydia pneumonia* (GSE12806), and *L. monocytogenes* infection for 2 hours in mice(GSE53145). (D) Induction of Rnd1 after infection with NDV and LM in HEK293 and human peripheral blood mononuclear cells (hPBMCs), respectively. (E) Rnd1 promoter was cloned upstream to luciferase gene. Resulting plasmid (Rnd1P) was transfected into HEK293 cells, and luciferase activity was analyzed 12 hours after treatment with IFN-α. RT-PCR data are means ± SEMs from triplicate samples of a single experiment and are representative of results from three independent experiments.

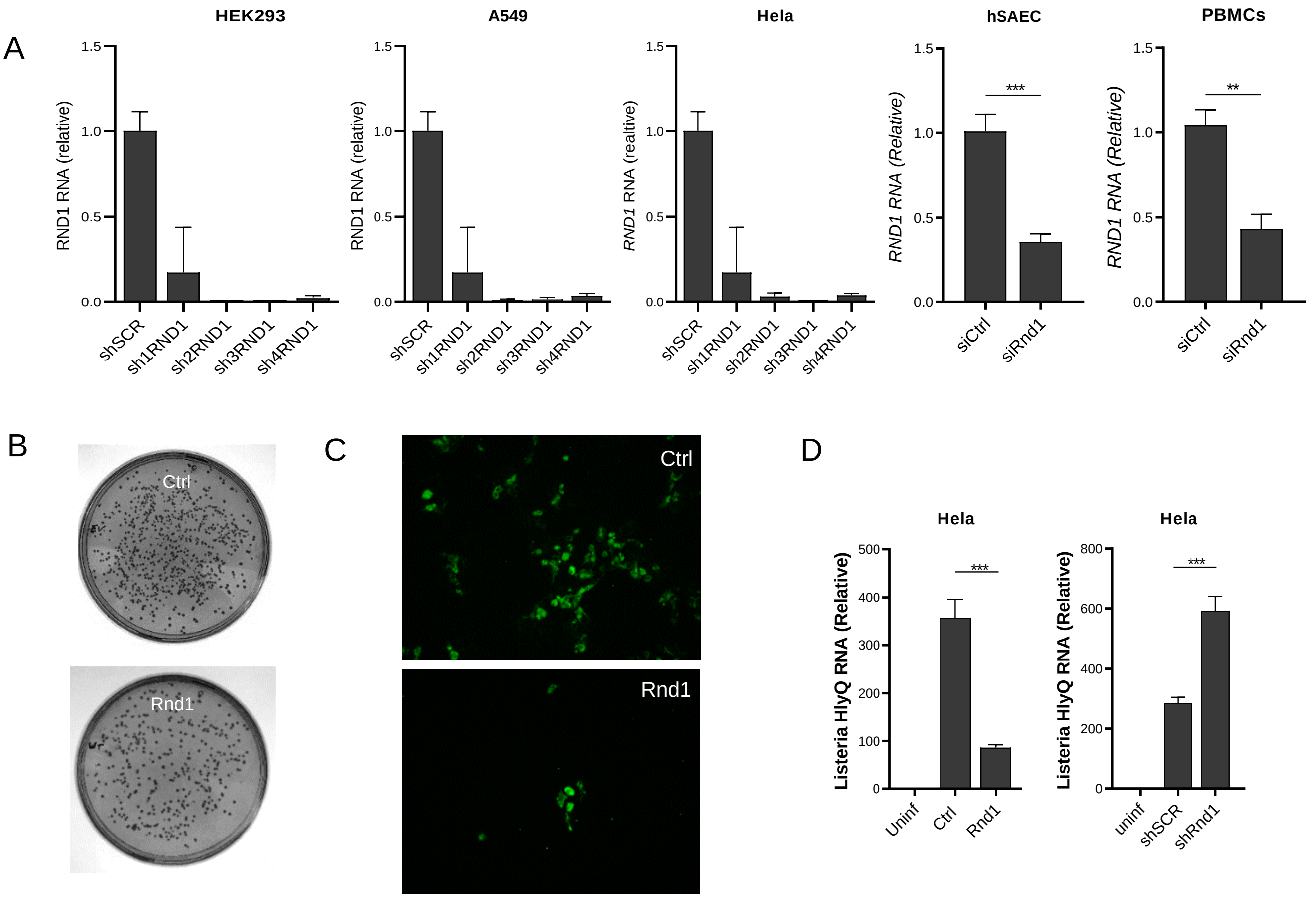

Supplementary Figure S2. (A) Efficiency of Rnd1 knockdown 48 hours after transfection of different shRNAs in HEK293, Hela and A549 cells or after 24 hours of transfection of siRNA targeting Rnd1 gene in primary hSAECs or PBMCs was analyzed using RT-PCR. Estimation of LM infection by (B) colony formation assay, and (C) microscopy. (D) Hela cells were infected with GFP-tagged L. monocytogens after Rnd1 was either overexpressed or knocked down. 24 hours post infection bacterial load was analyzed by RT-PCR analysis. Data are means ± SEMs from triplicate samples of a single experiment and are representative of results from three independent experiments.

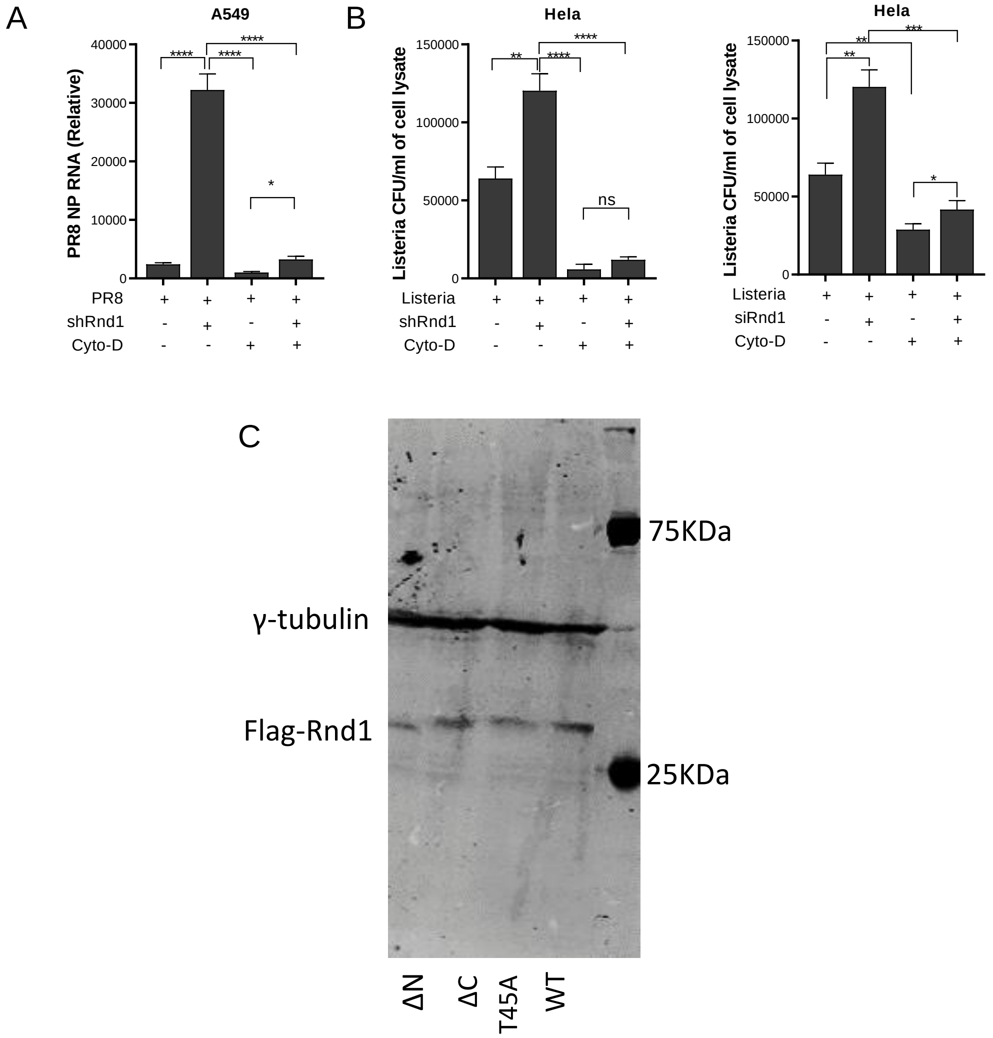

Supplementary Figure S3. (A) Rnd1 knockdown was performed in A549 cells using shRNA targeting Rnd1. Cells were treated with cytochalasin D for 30 minutes and subsequently infected with PR8. 24 hours post infection PR8 infection was analyzed by RT-PCR. (B) Rnd1 was knocked down in Hela cells using shRNA or siRNA targeting Rnd1. Cells were treated with cytochalasin D for 30 minutes and subsequently infected with LM. 24 hours post infection cells were lysed and LM infection was analyzed by colony formation assay. (C) Western blot for confirmation of expression of wild type Rnd1 and mutants. Blot was probed with gamma-tubulin and anti-flag antibody, data is representative of three independent experiments. Data are means ± SEMs from triplicate samples of a single experiment and are representative of results from three independent experiments.

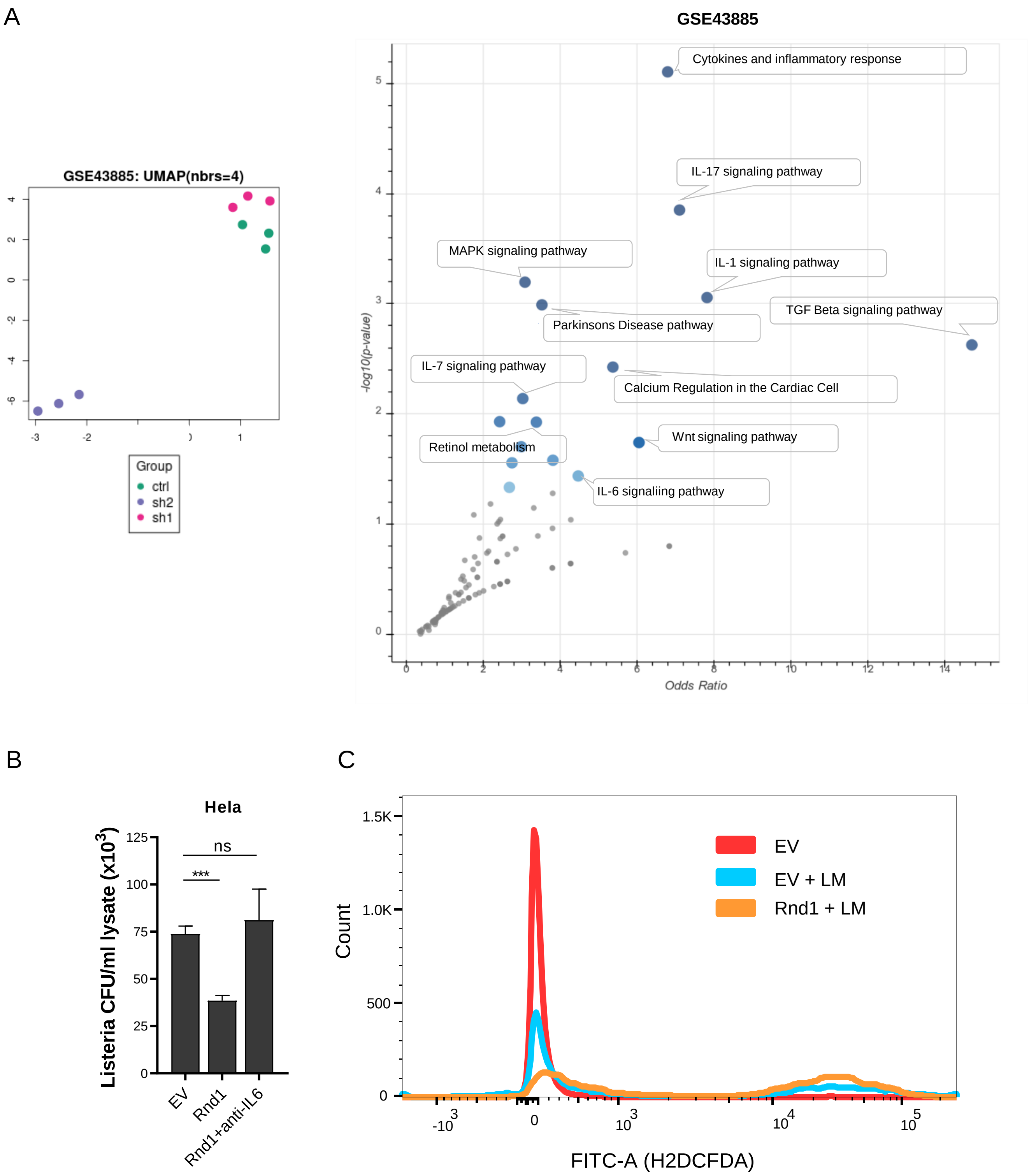

Supplementary Figure S4. (A) Reanalysis of publicly available dataset GSE43885, which involves RND1 knockdown in MCF10A cells. Uniform Manifold Approximation and Projection (UMAP) indicating that in terms gene expression all knockdown samples sufficiently differ from control samples (top). Gene ontological analysis of downregulated biological processes after Rnd1 knockdown (bottom). (B) Hela cells were transfected by with empty vector or Rnd1 plasmid, 24 hours post transfection cells were treated with anti-IL6 antibody for 3 hours. Cells were infected with *Listeria Monocytogenes* (LM) and bacterial load was analyzed 6 hours after infection data are means ± SEMs from triplicate samples of a single experiment and is indicative of three independent experiments. . (C) THP1 derived macrophage cells were electroporated with indicated plasmids and cells were infected with LM for 1 hour. Cells were stained with H2DCFDA for analysis of production of reactive oxygen species, data is representative of two independent experiments.

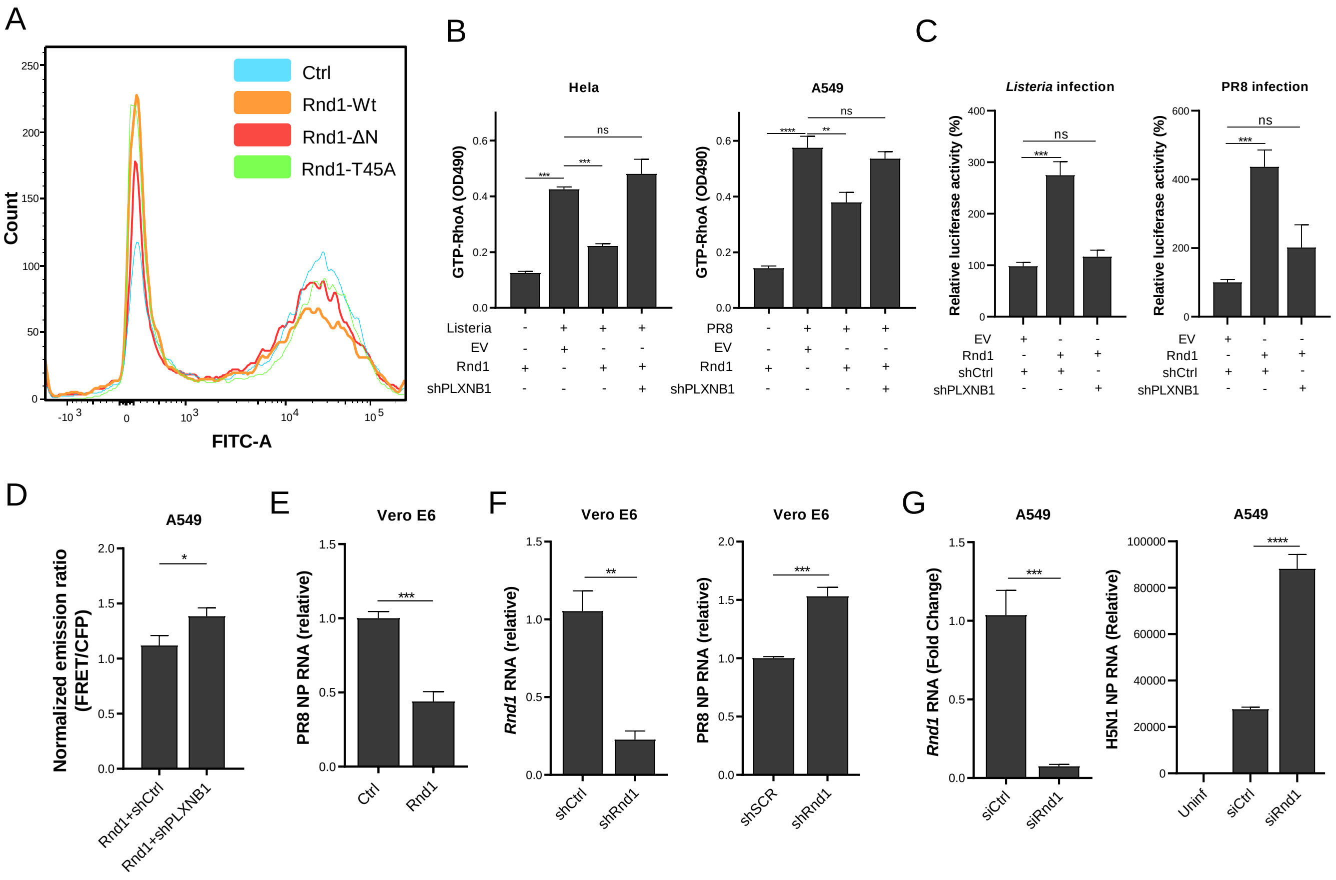

Supplementary Figure S5. (A) Hela cells were transfected either with vector backbone (Ctrl) or plasmids expressing Rnd1-Wt, Rnd1-ΔN, or Rnd1-T45A. 24 hours after transfection cells were infected with CFP labeled LM, followed by analysis of bacterial load by cytofluorometric analysis 24 hours post infection. (B) 24 hours after transfection with mentioned plasmids Hela cells were infected with LM and GTP bound RhoA was analyzed using ELISA. (C) HEK293 cells were transfected with NF-κB reporter plasmid, pRL-TK plasmid along with other indicated plasmids. Luciferase activity was analysed 24 hours post infection with LM or PR8 (D) Vero E6 cells were transfected either with control or Rnd1 specific shRNA, followed by infection with PR8 virus. 24 hours after transfections cells were harvested and Rnd1 or PR8 NP RNA was analyzed using gene specific primers by RT-PCR analysis. (E) A549 cells were transfected with either the control siRNA (siCtrl) or siRNA targeting Rnd1 (siRnd1). 24 hours after transfection cells were infected with H5N1 influenza virus. Total RNA was isolated and RT-PCR was performed to analyze Rnd1 knockdown efficiency and viral load. Total RNA was sent for RNA-Seq analysis. Data are means ± SEMs from triplicate samples of a single experiment and are representative of results from three independent experiments.

A

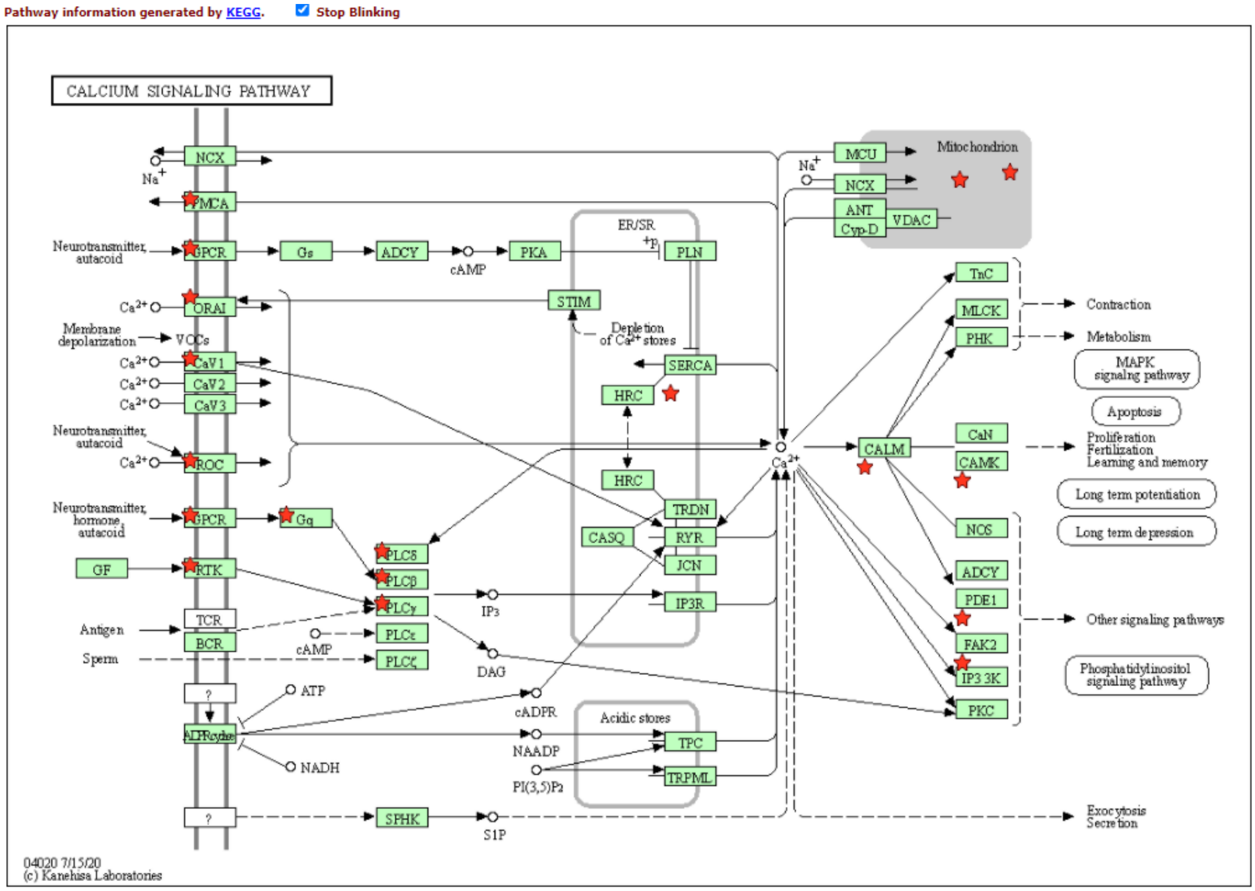

B

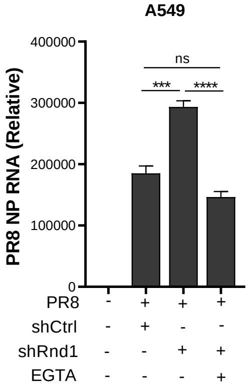

Supplementary Figure S6. (A) Detailed map of downregulated genes in calcium signaling pathway (generated by KEGG), genes found downregulated are highlighted. (B) A549 cells were transfected either with control shRNA or shRNA targeting Rnd1, 48 hours after transfection cells were infected with PR8 in presence or absence of EGTA. PR8 NP RNA was quantified by RT-PCR. RT-PCR data are means ± SEMs from triplicate samples of a single experiment and are representative of results from three independent experiments.

A

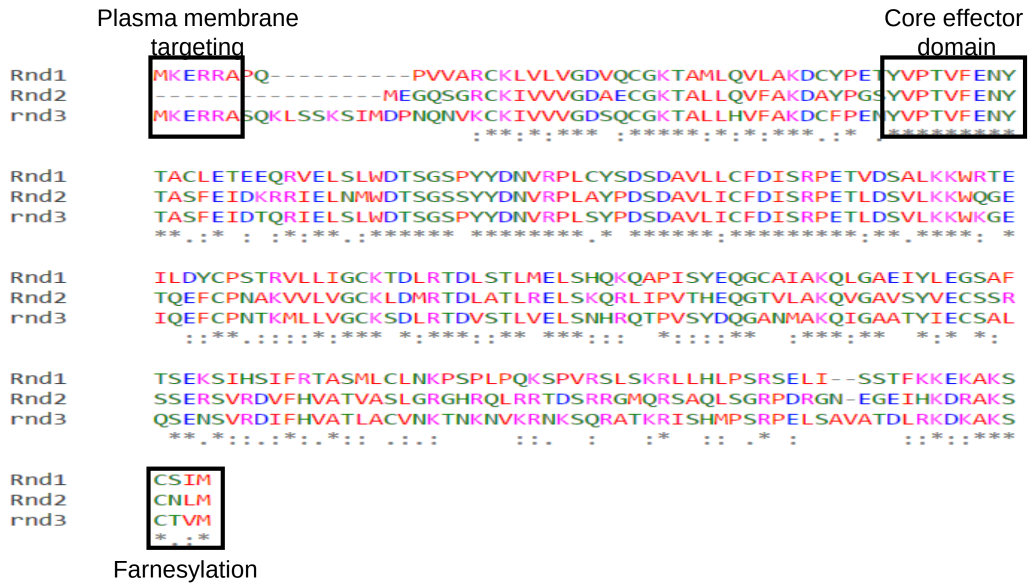

B

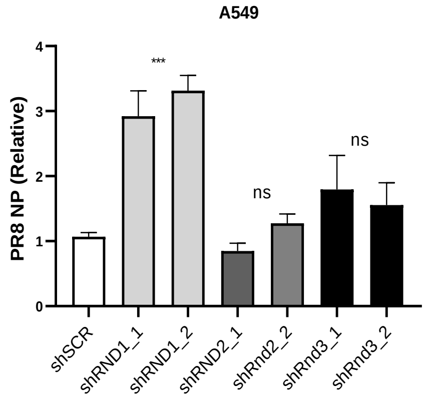

C

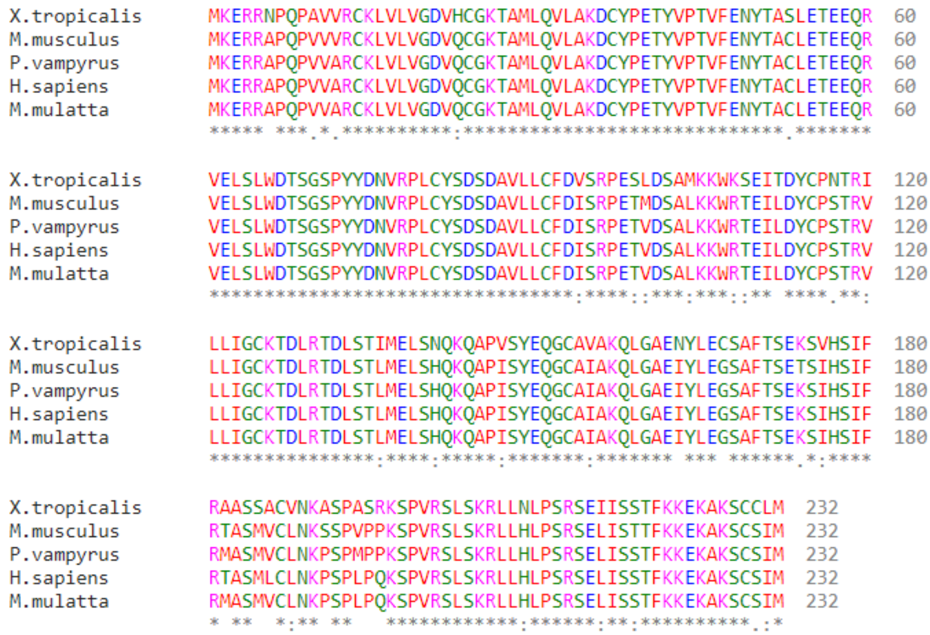

Supplementary Figure S7. (A) Comparison of Rnd1, Rnd2, and Rnd3 protein sequences. Functional domains of proteins have been highlighted. (B) A549 cells were transfected either with control shRNA (shSCR) or shRNA targeting Rnd1, Rnd2, or Rnd3. 48 hours after transfection cells were infected with PR8 followed by RT-PCR analysis of viral load 24 hours post infection. Data are means ± SEMs from triplicate samples of a single experiment and are representative of results from three independent experiments. (C) Comparison of RND1 protein sequences from diverse animal species Frog (*X. tropicalis*), mouse (*M. musculus*), Bat (*P.vampyrus*), humans (*H. sapiens*), and monkey (*M. mulatta*), indicating that Rnd1 sequence has been fairly conserved.

A

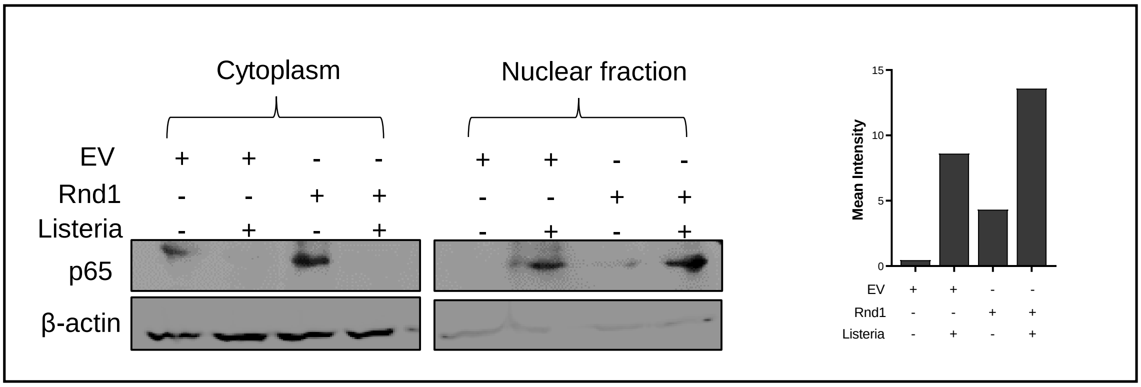
Cytoplasm
Nuclear fraction
EV
Rnd1
Listeria
p65
β-actin
Mean Intensity
15
10
5
0
EV
Rnd1
Listeria

B

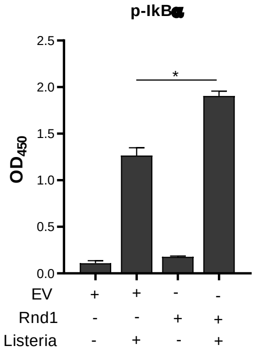
p-IkB
2.5
2.0
1.5
1.0
0.5
0.0
OD450
*
EV
Rnd1
Listeria

C

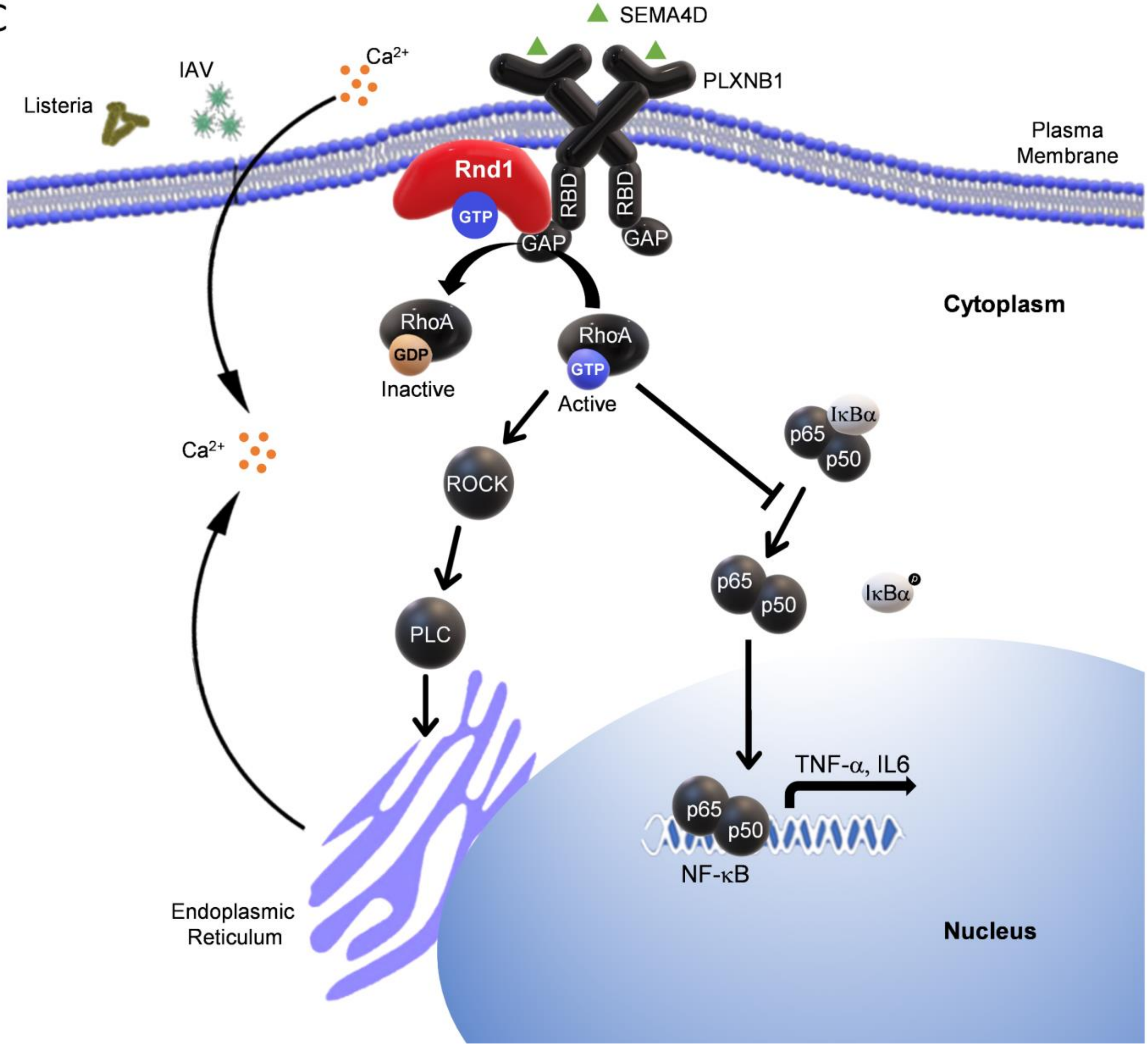
SEMA4D
PLXNB1
Listeria
IAV
Ca2+
Plasma Membrane
Rnd1
GTP
RBD
RBD
GAP
GAP
Cytoplasm
RhoA
GDP
Inactive
RhoA
GTP
Active
IκBα
p65
p50
ROCK
PLC
p65
p50
IκBα
Ca2+
TNF-α, IL6
p65
p50
NF-κB
Endoplasmic Reticulum
Nucleus

Supplementary Figure S8. (A) Western blot analysis of p65 in cytoplasmic and nuclear fractions, β-actin was used for analyzing purity of cytoplasmic and nuclear fractions. Data is representative of two independent experiments. Data is representative of two independent experiments. (B) Analysis of phosphorylated IkBα in cell lysates using ELISA. Data is representative of three independent experiments. Data is mean ± SEM three independent experiments. (C) Schematic representation of proposed mechanism of Rnd1 function during viral or bacterial infection. Rnd1 inactivate RhoA thus inhibiting intracellular calcium waves. Rnd1 also promoted NF-kB activation by inactivating its negative regulator, thus inducing pro-inflammatory cytokines.

Supplementary Table 1: List of primers

S. No.	Primer Name	Sequence
	**Cloning Primers**	
1	Rnd1-Promoter_Fw	GGTACCGCAACAAGAGCGAAACTCCATCTC
2	Rnd1- Promoter_Rv	AAGCTTGGTTGCAGTGTCCGCGGGACTT
3	Rnd1- ΔK_Fw	AAGCTTCCCCCCAGCCAGTCGTGG
4	Rnd1- ΔK_Rv	GGATCCCATAATGGAACAGCTTTTGGC
5	Rnd1- T45A_Fw	GCCCGCCGTGTTCGAAATTACACAGCC
6	Rnd1- T45A_Rv	ACGGCGGGCACATAGGTCTGG
7	RhoA_Fw	TTACGGATCCATGGCTGCCATCCGGAAG
8	RhoA_Rv	TTACCTCGAGTCACAAGACAAGGCACCCAG
	**RT-PCR Primers**	
9	Rnd1_Fw	ACTCTGCTACAGCGACTCG
10	Rnd1_Rv	CGGGTGCTGGGACAATAATC
11	18s rRNA_Fw	ATCACCATTATGCAGAATCCACG
12	18s rRNA_Rv	GACCTGGCTGTATTTTCCATCC
13	m18s rRNA_Fw	GTAACCCGTTGAACCCCATT
14	m18s rRNA_Rv	CCATCCAATCGGTAGTAGCG
15	mRnd1_Fw	GCTATCCCGAGACCTATGTGC
16	mRnd1_Rv	ACACGTGTGCTGGGACAGTA
17	mIL-6_Fw	ACCGCTATGAAGTTCCTCTCTGCA
18	mIL-6_Rv	AAGCCTCCGACTTGTGAAGTGGT
19	mTNF-α_Fw	ATGAGCACAGAAAGCATGA
20	mTNF-α_Rv	AGTAGACAGAAGAGCGTGGT
21	IFNβ_Fw	AGCTGAAGCAGTTCCAGAAG
22	IFNβ_Rv	AGTCTCATTCCAGCCAGTGC
23	PIP5K1B_Fw	CCAGGAATGGAAGGATGAGA
24	PIP5K1B_Rv	AATTGTGGTTGCCAAGGAAG
25	ROCK1_Fw	AGGTGATTGGTAGAGGTGCA
26	ROCK1_Rv	GCTGAACAACCCAAGGACTG
27	ROCK2_Fw	GGCGAGAATGTGATTGGTGG
28	ROCK2_Rv	TCCCAAGTCGTACCTCCCTA
29	PLCG1-Fw	AGCTGTGGTTCCCATCAAAC
30	PLCG1-Rv	CATGCTGATGGAGAAGACGA
31	Listeria_HlyQ-Fw	CATGGCACCACCAGCATCT
32	Listeria_HlyQ-Rv	ATCCGCGTGTTTCTTTTCGA
33	IL-6_Fw	CTCAGCCCTGAGAAAGGAGA
34	IL-6_Rv	CCAGGCAAGTCTCCTCATTG
35	IP-10_Fw	TGGCATTCAAGGAGTACCTCTC
36	IP-10_Rv	TGATCTCAACACGTGGACAAA
37	PLCG2-Fw	CAAAAAGGATTCGTCCGTGT
38	PLCG2-Rv	GTTTCACGCATGGTGTCATC
39	CAMK2D_Fw	ACTATCAACCCTGCCAAACG
40	CAMK2D_Rv	GGCTGCTGAGAAATTCCTTG
41	PR8-NP_Fw	GGAGGGGTGAGAATGGACGA
42	PR8-NP_Rv	GTCCATACACACAGGCAGGC
43	H5N1-NP_Fw	GGGCAGAACGTCTGACATGA
44	H5N1-NP_Rv	GGGTTCGTTGCCTTTTCGTC
45	NDV_Fw	TACAACAGGACATTGACCACTTTGCTCAC
46	NDV_Rv	TCGATCTTCCCAACTGCCACTGC
47	TNF-α_Fw	CAGAGGGAAGAGTTCCCCAGGGACC
48	TNF-α_Rv	CCTTGGTCTGGTAGGAGACGGCGATG
49	PLXNB1_Fw	TCTGCTCAGTGACCTGGTTG
50	PLXNB1_Rv	GTGTATTTGGCCTTGCCTGT
51	RhoA_Fw	AGGTAGAGTTGGCTTTGTGG
52	RhoA_Rv	CGTTGGGACAGAAATGCTTG
