# Supplementary figures and images for: Essential role of Rnd1 in innate immunity during viral and bacterial infections
Source: Cell Death Dis. 2022 Jun 2;13(6):520. doi: 10.1038/s41419-022-04954-y (PMC9161769; doi:10.1038/s41419-022-04954-y)

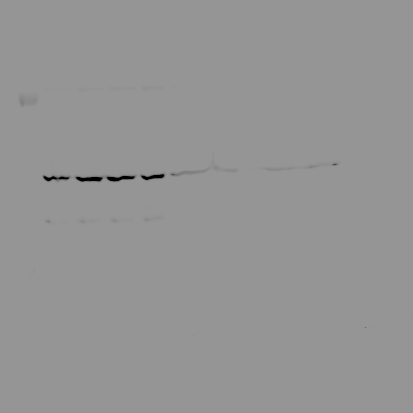

Supplement: Supplementary file 2 — Original Data File [file 41419_2022_4954_MOESM2_ESM.tif]

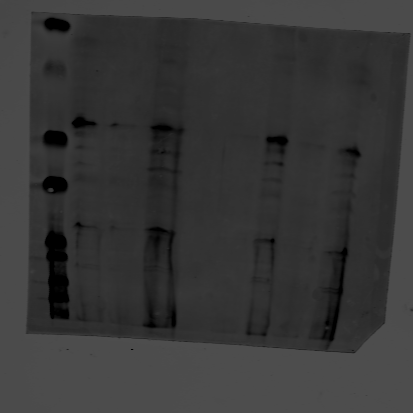

Supplement: Supplementary file 3 — Original Data File [file 41419_2022_4954_MOESM3_ESM.tif]

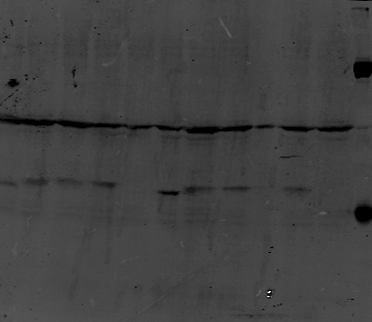

Supplement: Supplementary file 4 — Original Data File [file 41419_2022_4954_MOESM4_ESM.tif]

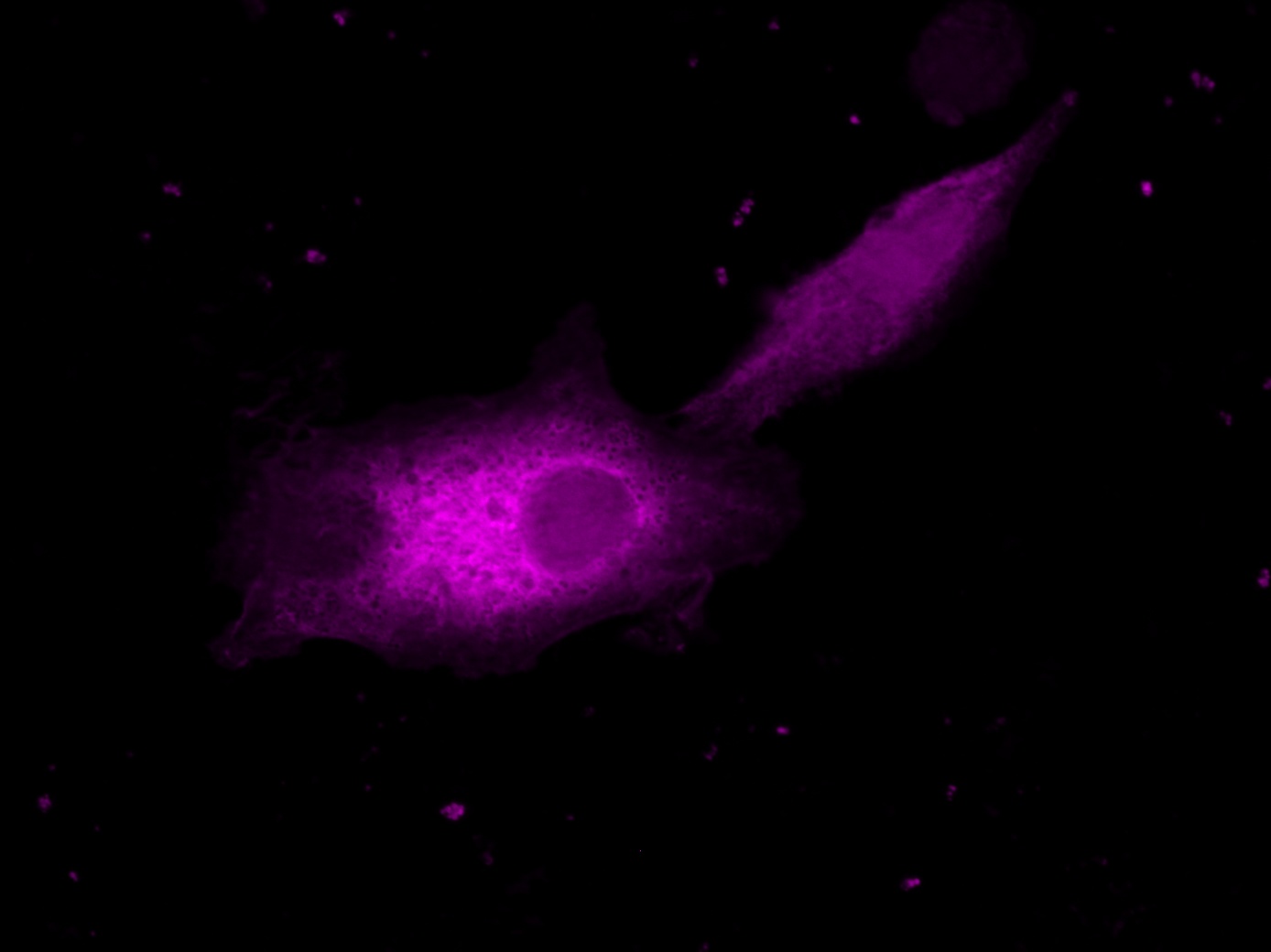

Supplement: Supplementary file 6 — Original Data File [file 41419_2022_4954_MOESM6_ESM.jpg]

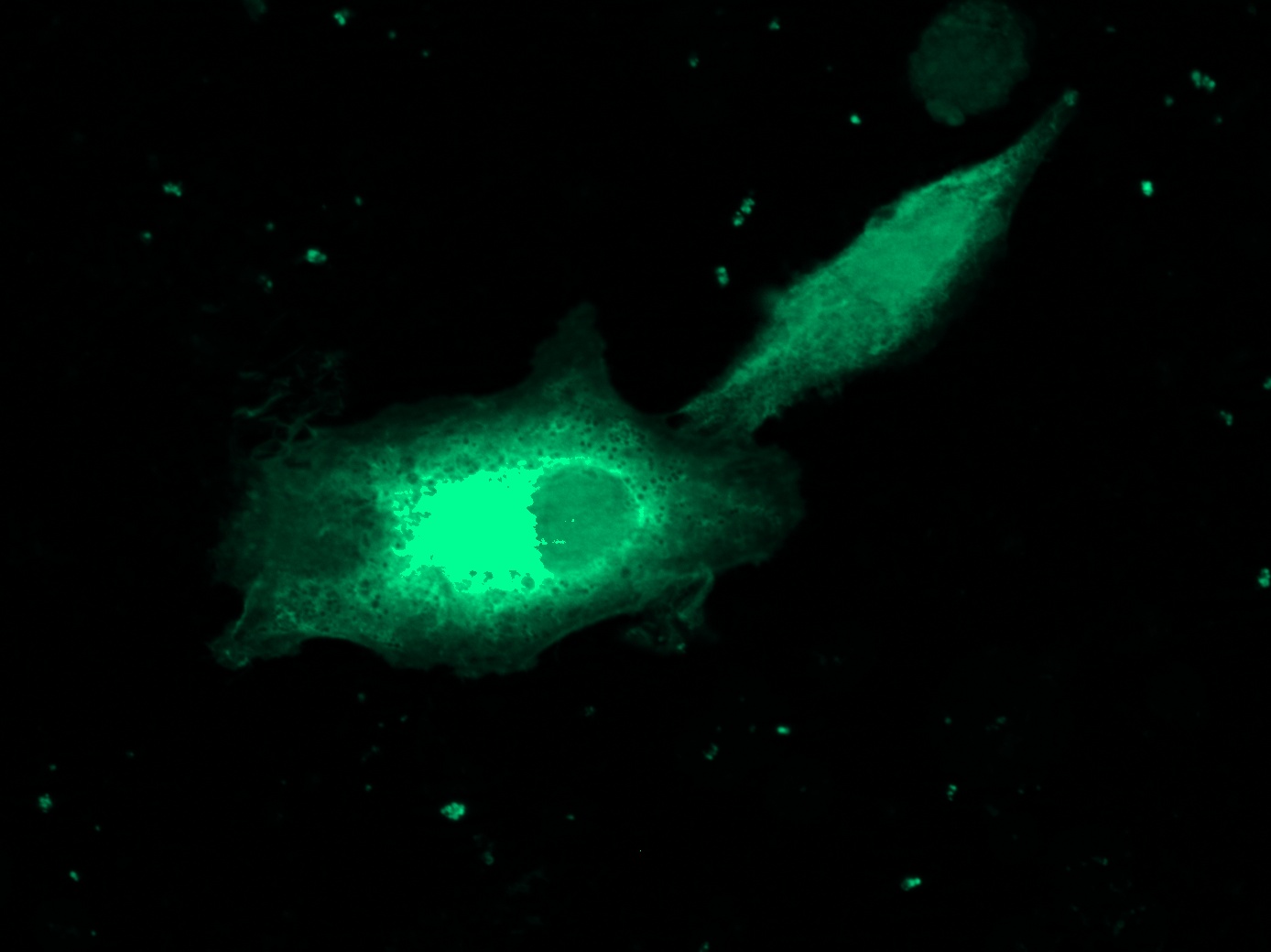

Supplement: Supplementary file 7 — Original Data File [file 41419_2022_4954_MOESM7_ESM.jpg]

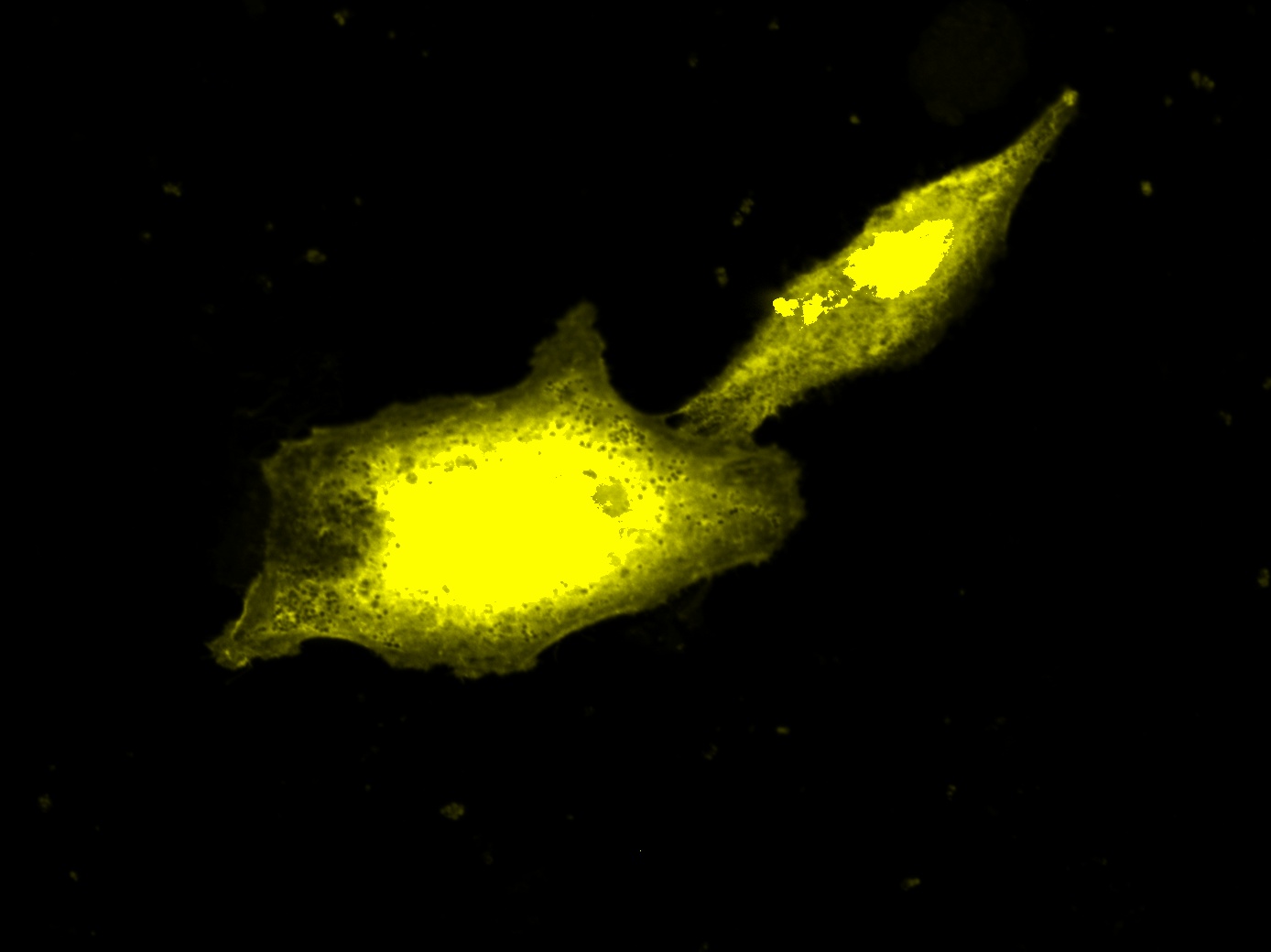

Supplement: Supplementary file 8 — Original Data File [file 41419_2022_4954_MOESM8_ESM.jpg]

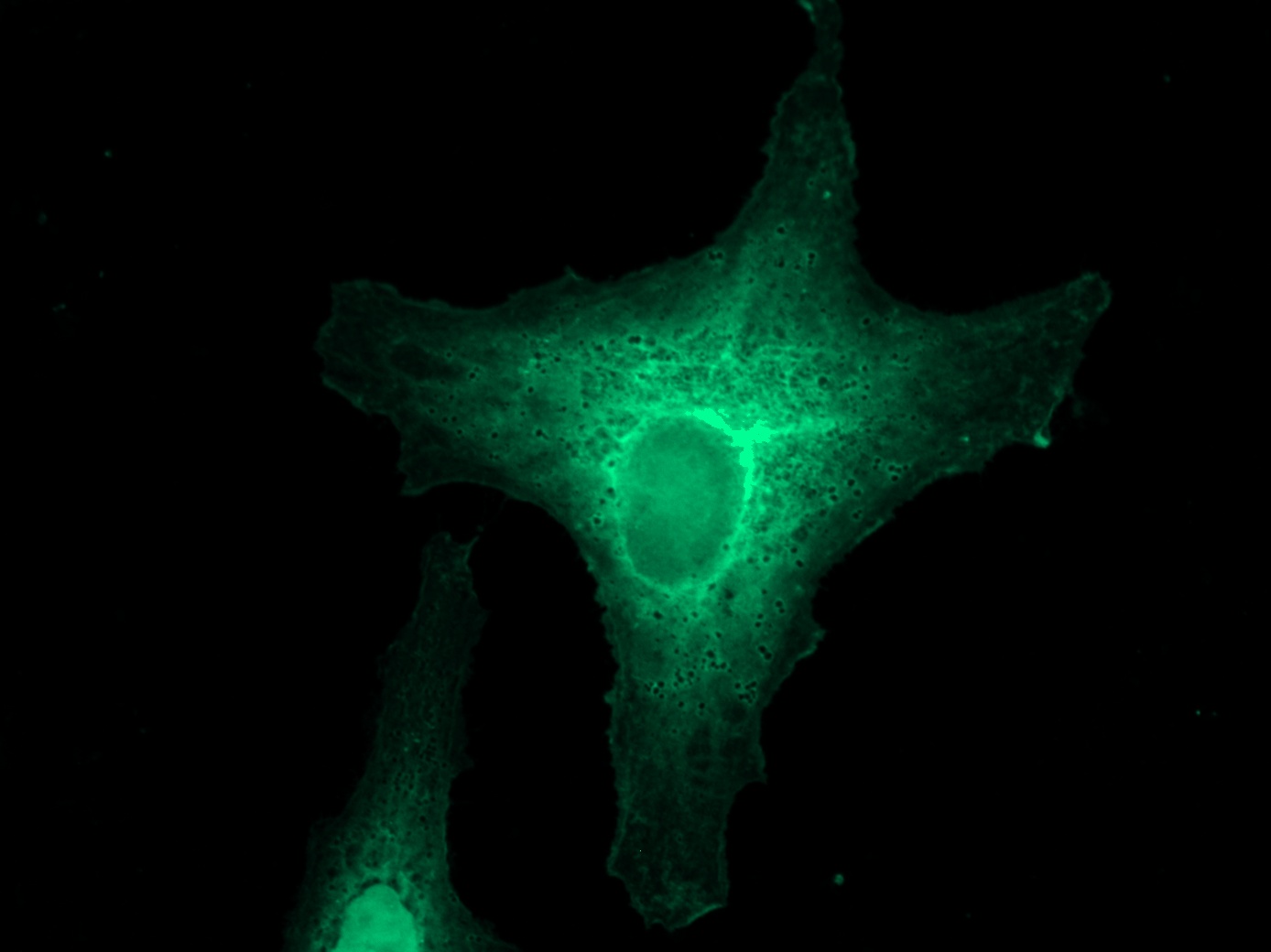

Supplement: Supplementary file 9 — Original Data File [file 41419_2022_4954_MOESM9_ESM.jpg]

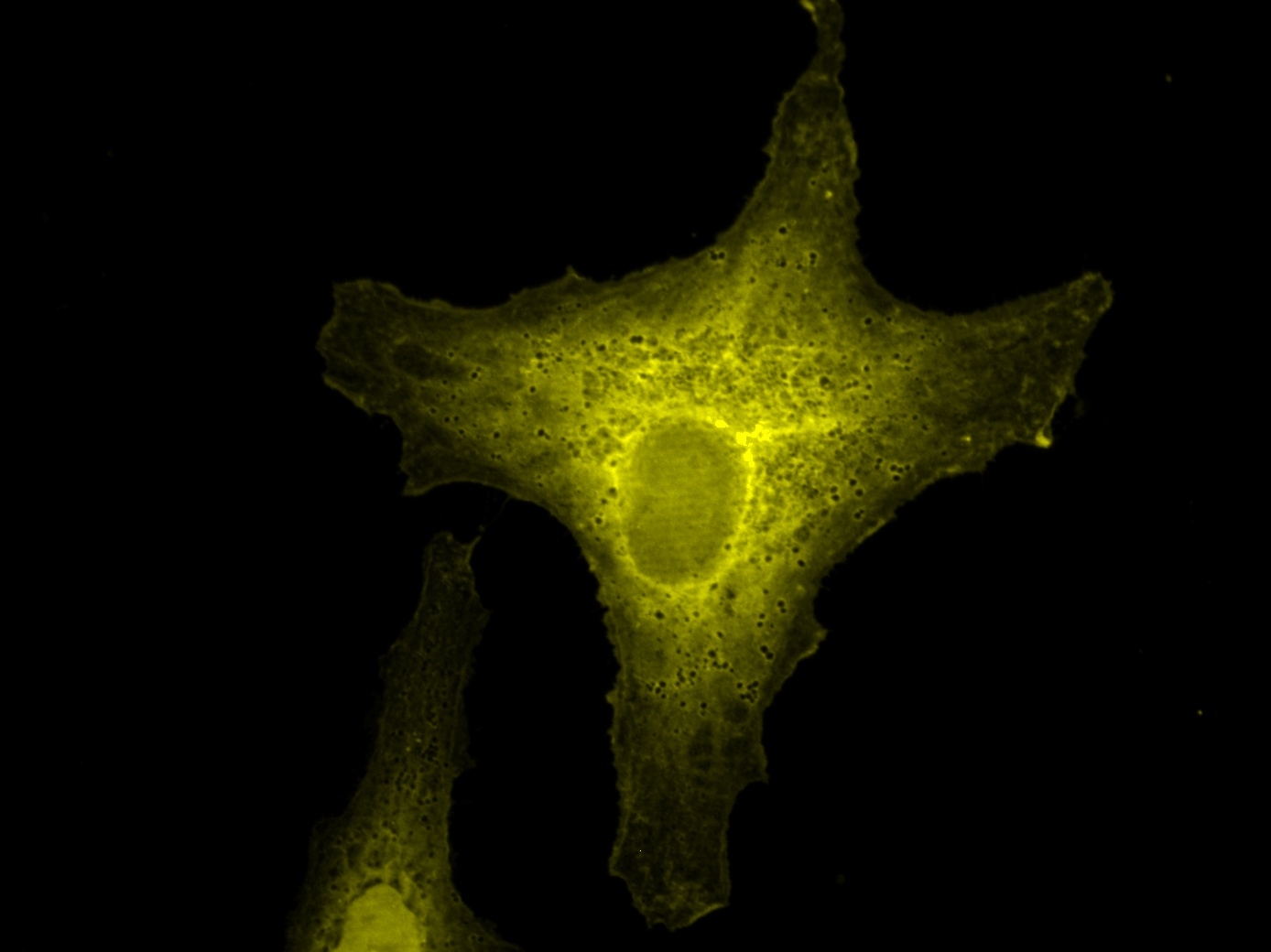

Supplement: Supplementary file 10 — Original Data File [file 41419_2022_4954_MOESM10_ESM.jpg]

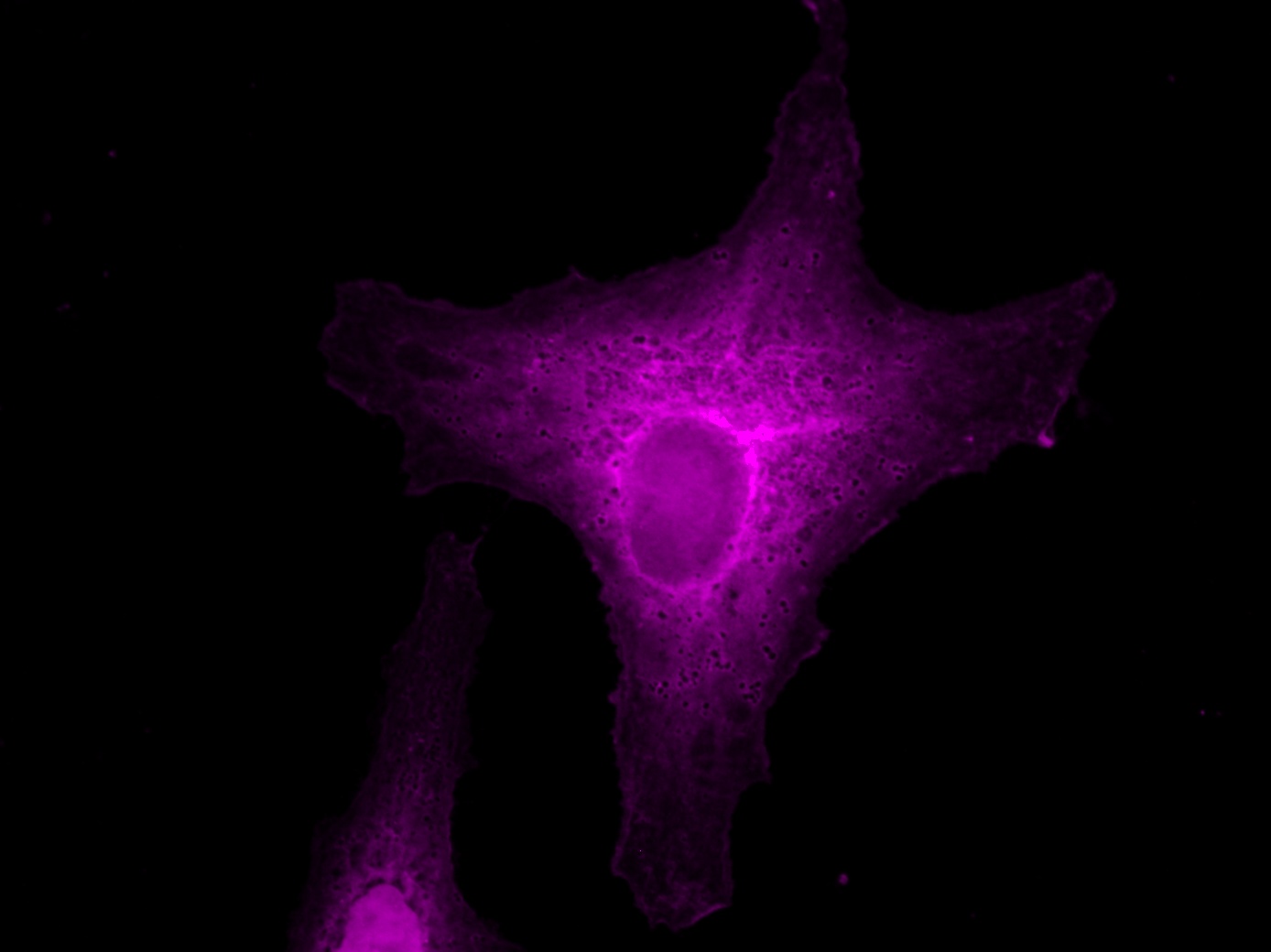

Supplement: Supplementary file 11 — Original Data File [file 41419_2022_4954_MOESM11_ESM.jpg]

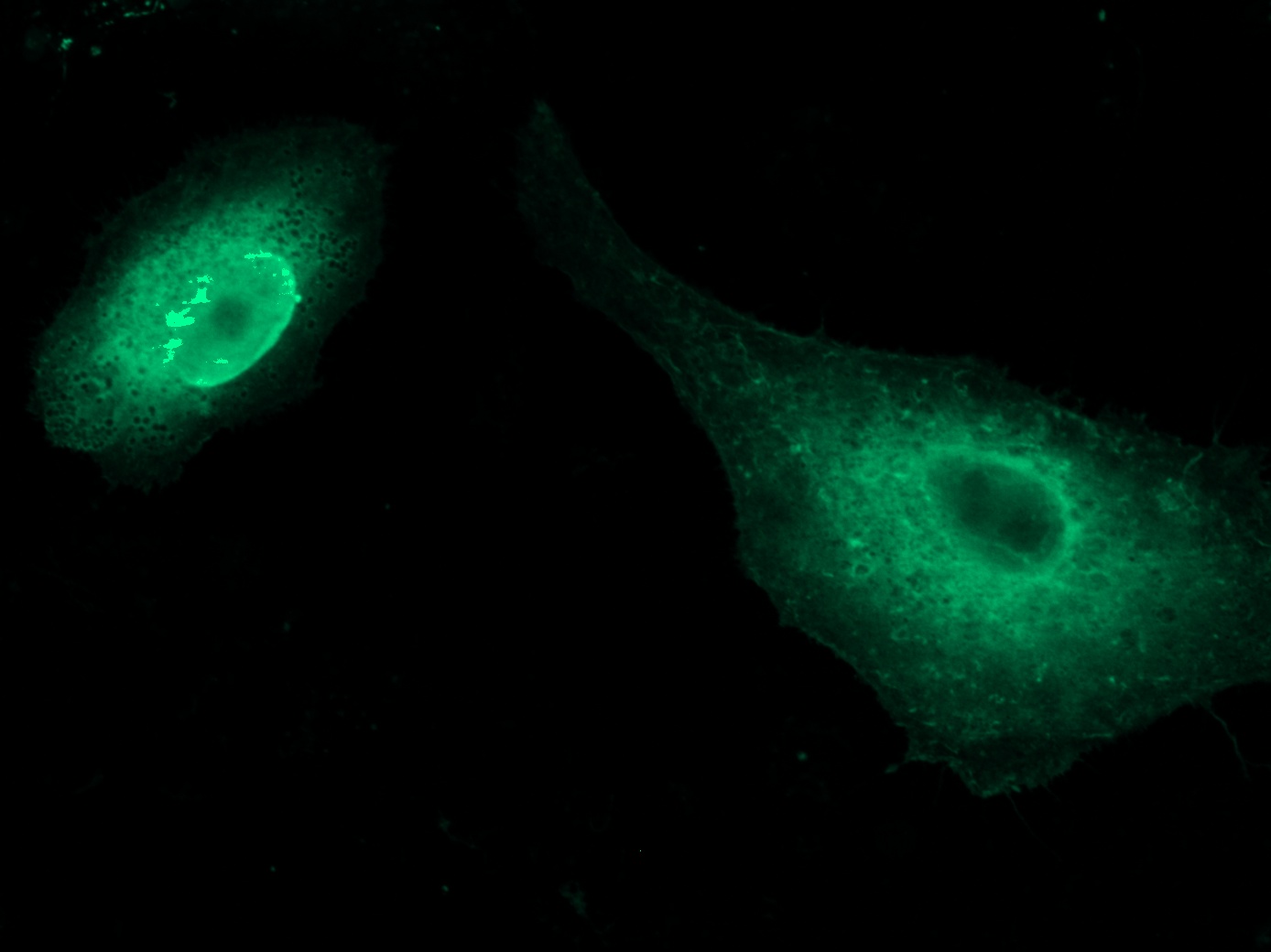

Supplement: Supplementary file 12 — Original Data File [file 41419_2022_4954_MOESM12_ESM.jpg]

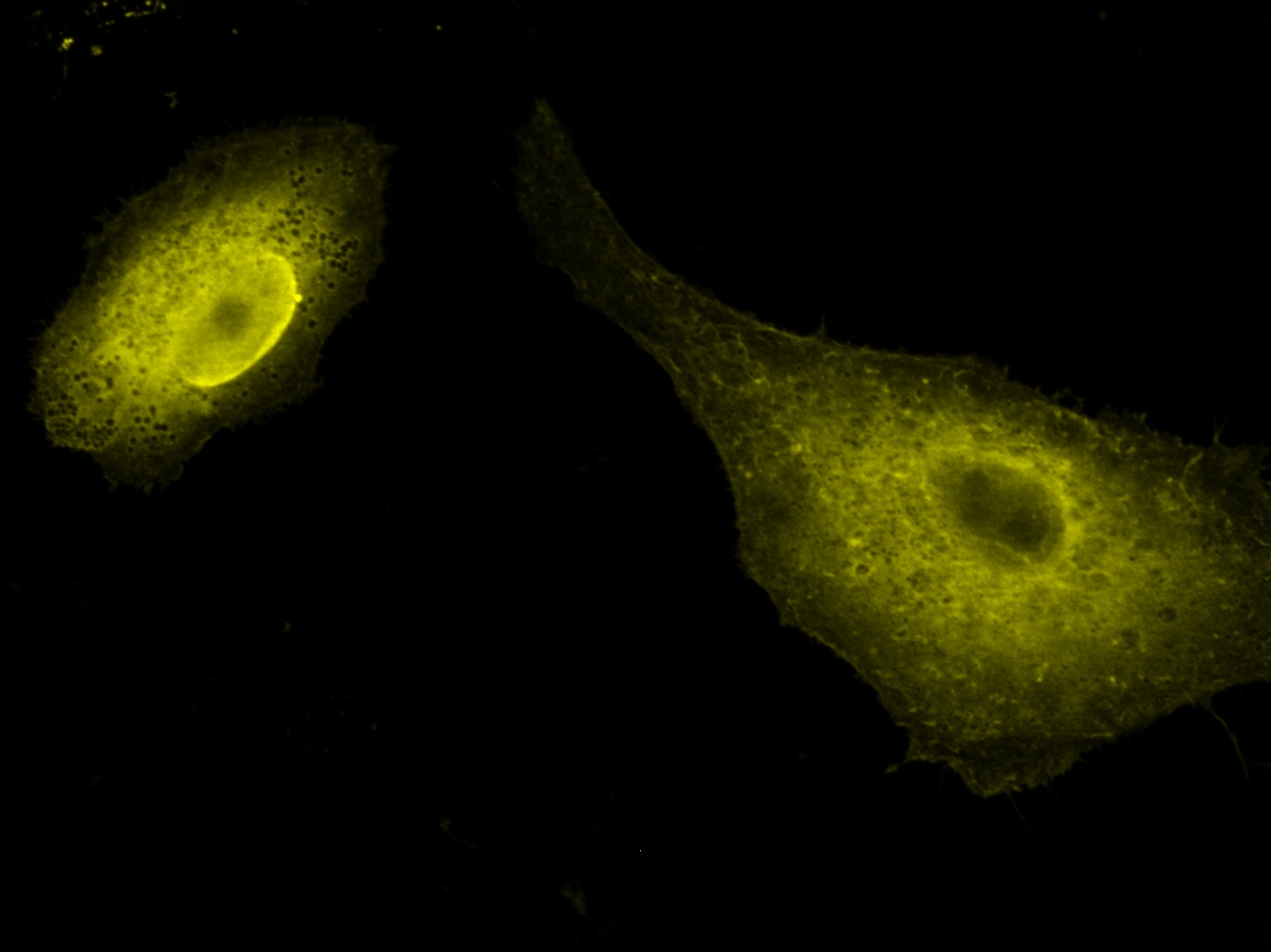

Supplement: Supplementary file 13 — Original Data File [file 41419_2022_4954_MOESM13_ESM.jpg]

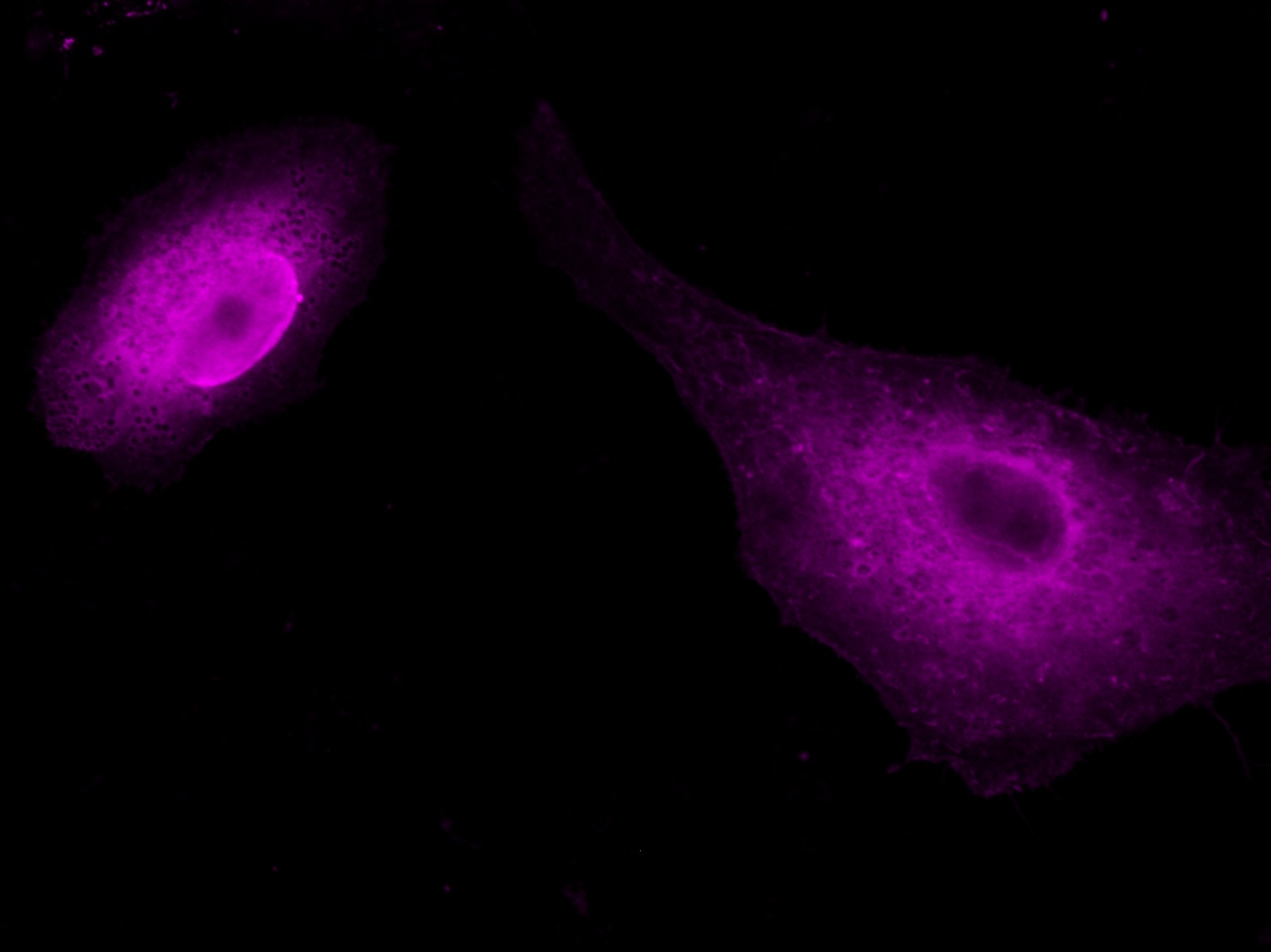

Supplement: Supplementary file 14 — Original Data File [file 41419_2022_4954_MOESM14_ESM.jpg]
